# Diagnostic performance of plasma pTau_217_, pTau_181_, Aβ_1-42_ and Aβ_1-40_ in the LUMIPULSE automated platform for the detection of Alzheimer disease

**DOI:** 10.1186/s13195-024-01513-9

**Published:** 2024-06-26

**Authors:** Javier Arranz, Nuole Zhu, Sara Rubio-Guerra, Íñigo Rodríguez-Baz, Rosa Ferrer, María Carmona-Iragui, Isabel Barroeta, Ignacio Illán-Gala, Miguel Santos-Santos, Juan Fortea, Alberto Lleó, Mireia Tondo, Daniel Alcolea

**Affiliations:** 1 Sant Pau Memory Unit, Department of Neurology, IR SANT PAU, Hospital de La Santa Creu I Sant Pau, C/Sant Quintí 89, 08041 Barcelona, Spain; 2 Department of Neurology, Unidad Alzheimer-Down, IR SANT PAU, Hospital de La Santa Creu I Sant Pau; Barcelona Down Medical Center, Fundació Catalana Síndrome de Down, Barcelona, Spain; 3 Universitat Autònoma de Barcelona, Barcelona, Spain; 4 Centro de Investigación Biomédica en Red en Enfermedades Neurodegenerativas, CIBERNED, Madrid, Spain; 5 Servei de Bioquímica I Biologia Molecular, IR SANT PAU, Hospital de La Santa Creu I Sant Pau, Universitat Autònoma de Barcelona, C/Sant Quintí 89, 08041 Barcelona, Spain; 6 Centro de Investigación Biomédica en Red en Diabetes y Enfermedades Metabólicas, CIBERDEM, Madrid, Spain

**Keywords:** Plasma, Biomarkers, Alzheimer, Blood, Amyloid, Tau

## Abstract

**Background:**

Recently developed blood markers for Alzheimer's disease (AD) detection have high accuracy but usually require ultra-sensitive analytic tools not commonly available in clinical laboratories, and their performance in clinical practice is unknown.

**Methods:**

We analyzed plasma samples from 290 consecutive participants that underwent lumbar puncture in routine clinical practice in a specialized memory clinic (66 cognitively unimpaired, 130 participants with mild cognitive impairment, and 94 with dementia). Participants were classified as amyloid positive (A +) or negative (A-) according to CSF Aβ_1–42_/Aβ_1–40_ ratio. Plasma pTau_217_, pTau_181_, Aβ_1–42_ and Aβ_1–40_ were measured in the fully-automated LUMIPULSE platform. We used linear regression to compare plasma biomarkers concentrations between A + and A- groups, evaluated Spearman’s correlation between plasma and CSF and performed ROC analyses to assess their diagnostic accuracy to detect brain amyloidosis as determined by CSF Aβ_1–42_/Aβ_1–40_ ratio. We analyzed the concordance of pTau_217_ with CSF amyloidosis.

**Results:**

Plasma pTau_217_ and pTau_181_ concentration were higher in A + than A- while the plasma Aβ_1–42_/Aβ_1–40_ ratio was lower in A + compared to A-. pTau_181_ and the Aβ_1–42_/Aβ_1–40_ ratio showed moderate correlation between plasma and CSF (Rho = 0.66 and 0.69, respectively). The areas under the ROC curve to discriminate A + from A- participants were 0.94 (95% CI 0.92–0.97) for pTau_217_, and 0.88 (95% CI 0.84–0.92) for both pTau_181_ and Aβ_1–42_/Aβ_1–40_. Chronic kidney disease (CKD) was related to increased plasma biomarker concentrations, but ratios were less affected. Plasma pTau_217_ had the highest fold change (× 3.2) and showed high predictive capability in discriminating A + from A-, having 4–7% misclassification rate. The global accuracy of plasma pTau_217_ using a two-threshold approach was robust in symptomatic groups, exceeding 90%.

**Conclusion:**

The evaluation of blood biomarkers on an automated platform exhibited high diagnostic accuracy for AD pathophysiology, and pTau_217_ showed excellent diagnostic accuracy to identify participants with AD in a consecutive sample representing the routine clinical practice in a specialized memory unit.

**Supplementary Information:**

The online version contains supplementary material available at 10.1186/s13195-024-01513-9.

## What is already known on this topic

Blood biomarkers have shown high accuracy to detect AD pathophysiology. The feasibility of those biomarkers in different platforms and the influence of comorbidities in their concentrations needs to be further studied.

## What this study adds

We analyzed the feasibility and diagnostic performance of blood AD biomarkers using a fully-automated platform in the setting of a memory clinic and assessed the impact of comorbidities on their diagnostic performance.

How this study might affect research, practice, or policy: The measurement of plasma AD biomarkers in an automated platform yields high accuracy to detect AD pathophysiology and would be easy to implement. Plasma pTau_217_ was the single most accurate marker for clinical implementation.

## Introduction

Early and accurate diagnosis is becoming an increasing priority with the recent developments of disease-modifying therapies for Alzheimer’s Disease (AD). Pathophysiological biomarkers in cerebrospinal fluid (CSF) and positron emission tomography (PET) imaging with amyloid and tau tracers have extensively proven to be useful to detect the disease pathophysiology but are either expensive and/or invasive [1], which can delay the diagnosis and access to treatment.

Measurement of AD biomarkers in blood through reliable high-throughput platforms would simplify the diagnostic process. This is now technically possible thanks to the development of sensitive technologies that can consistently quantify brain-derived molecules that are present in blood in very low concentrations [2–4]. Amyloid-β (Aβ) peptides and different isoforms of phosphorylated tau (pTau) in blood have shown high accuracy for detecting AD pathophysiology in previous research studies [5–12]*.* How all these plasma markers are affected by different comorbidities is also starting to be understood thanks to large well-characterized cohorts [13–15]. Thus, blood-based markers have the potential to be of great use in screening, early diagnosis, tracking progression, and ultimately, monitoring the efficacy of treatment [16–20]. Of all the plasma biomarkers evaluated, pTau_217_ has demonstrated a promising profile in identifying amyloidosis, showing the largest fold changes in symptomatic AD patients and the most predictive ability to identify cognitive decline [21–29]. In previous immunoassay studies CSF pTau_217_ showed better correlation with amyloid-PET and tau-PET than pTau_181_ [30]. Subsequent research has revealed comparable efficacy of pTau_217_ in both plasma and CSF for the identification of AD neuropathology and for distinguishing pathopshysiological AD from other neurodegenerative diseases [31–33]. However, most of the existing studies have assessed each of these plasma markers separately, measuring them on different platforms or through techniques not widely available in clinical laboratories, which limits their potential to be widely applied in the clinical routine. The implementation of blood AD markers in a fully-automated platform would facilitate their reproducibility and accessibility in clinical laboratories [34].

The fully-automated platform LUMIPULSE G, extensively used to measure CSF AD biomarkers in clinical laboratories world-wide, has recently launched developed assays to measure pTau_217_, pTau_181_, Aβ_1-42_ and Aβ_1-40_ in plasma. In this study, our aim was to assess the feasibility and diagnostic performance of pTau_217_, pTau_181_, Aβ_1-42_ and Aβ_1-40_ in plasma in the LUMIPULSE fully-automated platform in a cohort of well characterized consecutive individuals assessed in a memory clinic.

## Methods

### Study participants and clinical classification

**Fig. 1:**
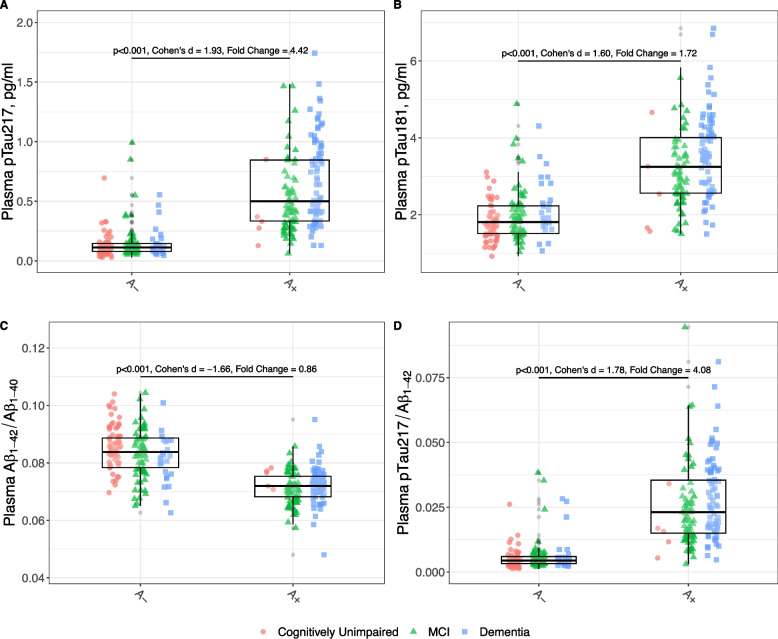
Levels of plasma biomarkers and their ratios according to the A status in CSF. All p-values are derived from multivariate linear model, adjusted for the effects of age, sex, *APOE ε4* status, chronic kidney disease stage, vascular risk factors and clinical stage. For better visualization, two outliers exceeding 2 pg/mL were excluded from the pTau_217_ boxplot for the A + group in the dementia stages. Not adjusted by other variables effect sizes are shown (Cohen’s d). Plasma biomarkers Cohen’s d and Fold Change calculated in log-transformed data. pTau_217_: phosphorylated tau 217, pTau_181_: phosphorylated tau 181. Aβ_1–42_: Amyloid β_1–42_. Aβ_1–40_: Amyloid β_1–40_. MCI: Mild cognitive impairment

We included all consecutive individuals who underwent lumbar puncture for the analysis of AD CSF biomarkers assessed at the Sant Pau Memory Unit (SPIN cohort, Barcelona, Spain) as part of their diagnostic work-up [35] between January 2021 and December 2021 (shown in flowchart in Supplementary Material (Fig. 1)**.** The study was approved by the Sant Pau Ethics Committee (Protocol code: EC/22/202/6880) following the standards for medical research in humans recommended by the Declaration of Helsinki. All participants or their legally authorized representative gave written informed consent to participate in biomarkers research studies.

At the time of CSF and plasma acquisition, participants had a diagnosis of dementia, mild cognitive impairment (MCI), or were cognitively unimpaired (CU). The clinical diagnosis was established after a thorough neurological and neuropsychological evaluation [35]. A more extensive description about how cognitively unimpaired participants were recruited and classified is provided in Supplementary Material (Text 1 & Fig. 1). To assess the impact of vascular risk factors and comorbidities, we collected information about the presence of high blood pressure, dyslipidemia, diabetes, obstructive sleep apnea and history of stroke. Participants were also classified according to the estimated glomerular filtrate rate (eGFR) in different stages (1–5) of chronic kidney disease (CKD) using CKD-EPI formula.

After a full evaluation that included analysis of AD CSF biomarkers, participants were classified according to their etiologic diagnosis, as Alzheimer disease (AD), other neurodegenerative diseases (OtherDem), not neurodegenerative diseases (OtherNotDeg) or CU. A proportion of participants' diagnosis was classified as “uncertain” as they had an unclear etiological diagnosis after a full initial evaluation and required clinical follow-up.

Cognitively unimpaired participants were patients with no evidence of cognitive impairment after a thorough neuropsychological evaluation and healthy volunteers interested in research.

### Sample collection and analysis

Blood samples were collected in EDTA-K2 tubes and subsequently centrifuged (2000 rpm × 10 min, 4ºC) within 2 h after extraction. Plasma was aliquoted and stored at -80ºC until analysis. CSF samples were obtained through lumbar puncture, and were also centrifuged, aliquoted and stored at -80ºC until analysis. Blood and CSF samples were collected simultaneously. Full protocol for CSF and blood sample collection in our center has been previously reported [35, 36].

All plasma samples were measured in the Lumipulse fully-automated platform G600II using commercially available kits (Fujirebio Europe, Ghent, Belgium) for pTau_181_, Aβ_1-42_ and Aβ_1-40_ between July and August 2022 with the same lot of reagents. Plasma pTau_217_ was analyzed between August and September 2023 in another aliquot of the same samples using a novel assay recently developed by Fujirebio. On the day of the analysis, plasma samples were brought to room temperature, mixed thoroughly, centrifuged for 5 min at 2000 g, and subsequently transferred to specific cuvettes for analysis in the Lumipulse platform.

CSF markers Aβ_1–42_, Aβ_1–40_, pTau_181_ and tTau were used in the diagnostic assessment of patients and measured in routine runs scheduled twice a month throughout 2021 following previously reported methods [37]. According to CSF markers, all participants were classified as amyloid positive (A + , CSF Aβ_1–42_/Aβ_1–40_ < 0.062) or negative (A-), and as tau positive (T + , pTau_181_ > 63 pg/mL) or negative (T-). Validation of these cutoff values has been described elsewhere [37]. To validate that our main findings would not be overly influenced by analytical error in CSF measurements, we performed additional sensitivity analyses excluding participants ± 10% of the CSF cutoff points (Supplementary Material, Figs. 1–3).

DNA was extracted from full blood using standard procedures, and *APOE* was genotyped following previously reported methods [35]. Briefly, direct DNA sequencing of exon 4 was performed routinely for all participants in the SPIN cohort, followed by visual analysis of the resulting electropherogram to identify the two coding polymorphisms that encode the three possible *APOE* isoforms.

### Statistical analysis

Data normality was assessed with the Shapiro–Wilk test. Non-normally distributed variables were log-transformed when necessary. Kruskal–Wallis and Wilcoxon rank sum test were used in continuous variables that were not normal distributed. Linear regression models and ANCOVA adjusted by age and sex were performed for group comparison. We used Chi Square test to assess differences in categorical variables and Fisher’s exact test in group comparisons with small number of observations, and Spearman test to assess the correlation between plasma and CSF markers.

Diagnostic accuracy of plasma biomarkers was assessed through receiver operating characteristic (ROC) analysis. We calculated the areas under the curve (AUC) of individual markers and that of logistic regression models that combined them with each other and with clinical variables. A basic model that included Age, Sex and *APOEε4* status was used as a reference to assess the added diagnostic value of plasma markers. We compared the accuracy of individual markers and regression models using DeLong's test adjusted by multiple comparisons using Bonferroni method. We evaluated the sensitivity, specificity, and Youden’s J index of a range of cutoffs to discriminate A + from A- participants. We analyzed the concordance of pTau_217_ with CSF amyloidosis. We followed a previously reported approach [38] to stratify our cohort in low, medium, and high risk of having CSF amyloidosis. Using predictive models, according to the risk of the participants of being A + , we selected conservative (97.5% sensitivity/specificity) and more lenient (95% sens/spec) cutoffs. We bootstrapped to assess the cutoff robustness. All tests were performed in R statistical software version 4.2.1. Alpha threshold was set at 0.05 for all analysis.

## Results

### Study participants and clinical classification

We included 290 participants who were syndromically classified as cognitively unimpaired (CU, *n* = 66), having mild cognitive impairment (MCI, *n* = 130) or a clinical diagnosis of dementia (*n* = 94). Table 1 shows the etiologic diagnoses in each category, main demographic characteristics, and biomarker measures in each group. CU participants were younger than those with MCI (*p* < 0.001) and those with dementia (*p* < 0.001). There were more female participants (62%). The proportion of A + , A + T + and *APOEε4* positive increased according to the clinical stage. More extensive demographics details, including stratification by clinical diagnosis or A status, can be found in Supplementary Material (Tables 1 & 2).

**Table 1:** Demographics and plasma biomarker concentrations

	**Cognitively Unimpaired, *N* = 66**	**MCI, *N* = 130**	**Dementia, *N* = 94**	***P* value**
**Age**	57 (12)	73 (11)	75 (9)	** < 0.001**
**Sex (Female)**	46 (70%)	77 (59%)	57 (61%)	0.3
**MMSE**	29.0 (1.0)	26.0 (4.0)	22.0 (5.8)	** < 0.001**
**APOE***ε4*	15 (23%)	32 (25%)	36 (38%)	**0.042**
**Plasma pTau**_**217**_ (pg/mL)	0.11 (0.08)	0.23 (0.35)	0.46 (0.63)	** < 0.001**
**Plasma pTau**_**181**_ (pg/mL)	1.71 (0.55)	2.32 (1.28)	3.35 (1.86)	** < 0.001**
**Plasma Aβ**_**1-42**_ (pg/mL)	25.0 (4.6)	23.0 (5.0)	24.6 (6.2)	**0.028**
**Plasma Aβ**_**1-40**_ (pg/mL)	290 (65)	301 (66)	319 (72)	** < 0.001**
**Plasma Aβ**_**1-42**_**/Aβ**_**1-40**_	0.085 (0.010)	0.076 (0.014)	0.074 (0.008)	** < 0.001**
**A**				** < 0.001**
A +	5 (7.6%)	66 (51%)	69 (73%)	
**AT**				** < 0.001**
A-T-	61 (92%)	64 (49%)	25 (27%)	
A + T-	2 (3.0%)	17 (13%)	12 (13%)	
A + T +	3 (4.5%)	49 (38%)	57 (61%)	
**Etiology**				** < 0.001**
CU	56 (85%)	0 (0%)	0 (0%)	
AD	4 (6.1%)	43 (33%)	50 (53%)	
OtherNotDeg	1 (1.5%)	37 (28%)	11 (12%)	
OtherDem	3 (4.5%)	18 (14%)	20 (21%)	
Uncertain	2 (3.0%)	32 (25%)	13 (14%)	
**VRF**	39 (60%)	102 (82%)	75 (85%)	** < 0.001**
**HBP**	24 (37%)	71 (57%)	49 (56%)	**0.021**
**DM**	11 (17%)	28 (23%)	20 (23%)	0.6
**DLP**	21 (32%)	64 (52%)	55 (63%)	**0.001**
**eGFR**_(mL/min/1.73m3_)				** < 0.001**
> 90	36 (64%)	29 (23%)	13 (14%)	
60–90	18 (32%)	91 (71%)	68 (72%)	
< 60	2 (3.6%)	8 (6.3%)	13 (14%)	

Median (IQR); n (%)

Kruskal–Wallis rank sum test was used to compare continuous variables that were not normally distributed; Pearson’s Chi-squared test was used to compare categorical variables; Fisher’s exact test was used to compare categorical variables with small number of observations

Unless otherwise specified, values are presented as median (IQR)

*CU* Cognitively Unimpaired, *MCI* Mild cognitive impairment, *AD* Alzheimer disease, *OtherNotDeg* Other not degenerative, *OtherDem* Other diseases, *VRF* one or more vascular risk factors, *HBP* High blood pressure, *DM* Diabetes mellitus, *DLP* Dyslipidemia, *eGFR* Estimated glomerular filtration rate

### Measures of pTau_217,_ pTau_181_, Aβ_1–42_ and Aβ_1–40_ in plasma

All plasma measures for pTau_217_, pTau_181_, Aβ_1–42_ and Aβ_1–40_ were above their lower limit of quantification. The plasma concentration ranges in the study were 0.03 to 2.84 for pTau_217_, 0.92 to 9.63 pg/mL for pTau_181_, 14.06 to 50.84 pg/mL for Aβ_1–42_ and 180.41 to 569.89 pg/mL for Aβ_1–40_. Inter-assay coefficients of variation are shown in Supplementary Material (Table 3).

### Correlation between plasma and CSF biomarkers

As per inclusion criteria, all participants had CSF biomarkers measures, and we explored the correlation between both matrices. The correlation between plasma and CSF was moderate for pTau_181_ (Rho = 0.66, *p* < 0.001) and low for Aβ_1–42_ (Rho = 0.26, *p* = 0.007), and Aβ_1–40_ (Rho 0.11, *p* = 0.06). When using ratios, the correlation was moderate for Aβ_1–42_/Aβ_1–40_ (Rho = 0.69, *p* < 0.001). The correlation between plasma pTau_217_ and CSF pTau_181_ was high (Rho = 0.75, *p* < 0.001). Both plasma pTau_217_ and pTau_181_ correlated with age (Rho = 0.42, *p* < 0.001 and Rho = 0.45, *p* < 0.001 respectively). Detailed correlations within clinical subgroups and partial correlations are shown in Supplementary Material (Figs. 4 and 5).

### Association between plasma biomarkers and amyloid status in CSF

We assessed the differences in plasma biomarkers between CSF amyloid positive and amyloid negative individuals considering other variables in a multivariate model. We studied the effect of age, sex, *APOE* status (*APOE ε4* +), renal function measured by the estimated glomerular filtration rate (eGFR), vascular risk factors (presence of at least one of the following: high blood pressure, diabetes mellitus, dyslipidemia, history of stroke, obstructive sleep apnea with CPAP) and clinical status (CU, MCI and Dementia). As shown in Fig. 1, the log transformed multivariate model confirmed that the A + group had higher plasma concentrations of pTau_217_ (fold-change 4.42, *p* < 0.001) and pTau_181_ (fold-change 1.72, *p* < 0.001) compared to the A- group. Similar results were seen using the ratios pTau_217_/Aβ_1–42_ and Aβ_1–42_/Aβ_1–40._ The plasma pTau_217_/Aβ_1–42_ ratio was higher in A + compared to A- (fold-change 4.08, *p* < 0.001). The plasma Aβ_1–42_/Aβ_1–40_ ratio was lower in A + compared to A- (fold-change 0.86, *p* < 0.001). We found lower plasma concentrations of Aβ_1–42_ in A + compared to the A- group (fold-change 0.92, *p* < 0.001) and no differences in Aβ_1–40_ concentrations between A status (not shown). To assess the influence of the values close to CSF Aβ_1–42_/Aβ_1–40_ cutoff, we conducted a sensitivity analysis by excluding participants with values close to the cutoff point (± 10%) (Supplementary Material, Figs. 1– 3). We found no significant influence of these observations in our analysis.

### Effect of other variables on plasma biomarkers

**Fig. 2:**
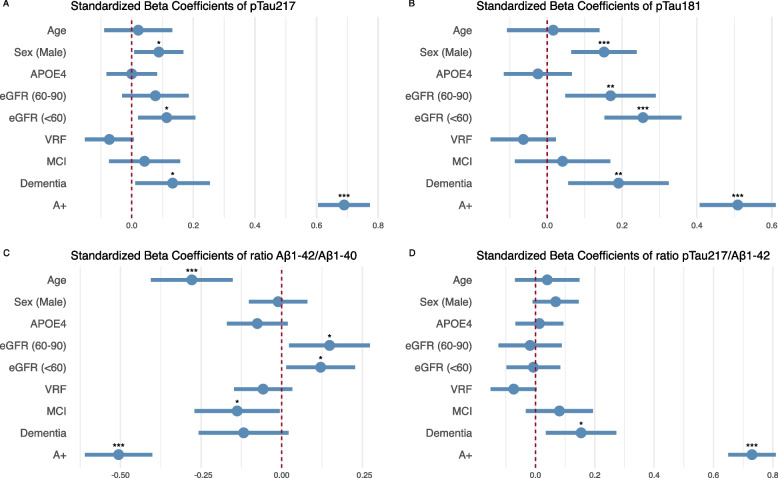
Effect of different variables on plasma pTau_217_, pTau_181_, Aβ_1–42_/Aβ_1–40_ and pTau_217_/ Aβ_1–42._ Dots and bars represent the standardized beta coefficients of each variable in a multivariate regression model. Lines represent the 95% confidence interval for each standardized beta coefficient. Red vertical dashed lines indicate a null effect. We can see the effect size of A positivity adjusted by other variables. pTau_217_: phosphorylated tau 217. pTau_181_: phosphorylated tau 181. Aβ_1–42_: Amyloid β_1–42_. Aβ_1–40_: Amyloid β_1–40_. MCI: mild cognitive impairment. VRF: vascular risk factors. eGFR: estimated glomerular filtration rate

We assessed whether log transformed plasma pTau_217_, pTau_181_, Aβ_1-42_ and Aβ_1-40_ were affected by other variables in the multivariate model. As seen in Fig. 2, amyloid positivity was the variable with the largest effect on all plasma markers. We also observed that decreased renal function was associated with higher concentrations of pTau_217_ (*p* = 0.019) and pTau_181_ (*p* < 0.001) and higher Aβ_1–42_/Aβ_1–40_ ratio (*p* < 0.001). Male sex was associated with higher pTau_217_ (*p* = 0.033) and pTau_181_ (*p* = 0.0009), and age was associated with lower Aβ_1–42_/Aβ_1–40_ ratio_._ Our model had and adjusted R^2^ value of 0.62 for pTau_217_, 0.52 for pTau_181_ and 0.48 for Aβ_1–42_/Aβ_1–40_. All the presented coefficients were obtained from the model in which all the listed variables were included. Complete forest plot shown in Supplementary Material** (**Fig. 6)**.**

To further investigate the association of pTau_217_ and pTau_181_ with renal function, we performed a subanalysis stratifying by estimated glomerular filtration rate. We found that pTau_181_ concentration in plasma was higher as renal function decreased (< 60 vs 60-90 mL/min/1.73m^2^ and < 60 vs. > 90 mL/min/1.73m^2^, *p* < 0.001). Aβ_1–42_ and Aβ_1–40_ concentrations in plasma were also higher as renal function decreased (*p* < 0.001). We also observed marginally significant differences in pTau_217_ concentrations in plasma samples from patients with low renal function (< 60 mL/min/1.73m^2^) compared to those with normal renal function (> 90 mL/min/1.73m^2^, *p* = 0.047). However, those differences were lost when using the Aβ_1–42_/Aβ_1–40_ or the pTau_217_/Aβ_1–42_ ratios.

### Diagnostic accuracy of plasma biomarkers and their combinations for the discrimination of A + from A-

**Fig. 3:**
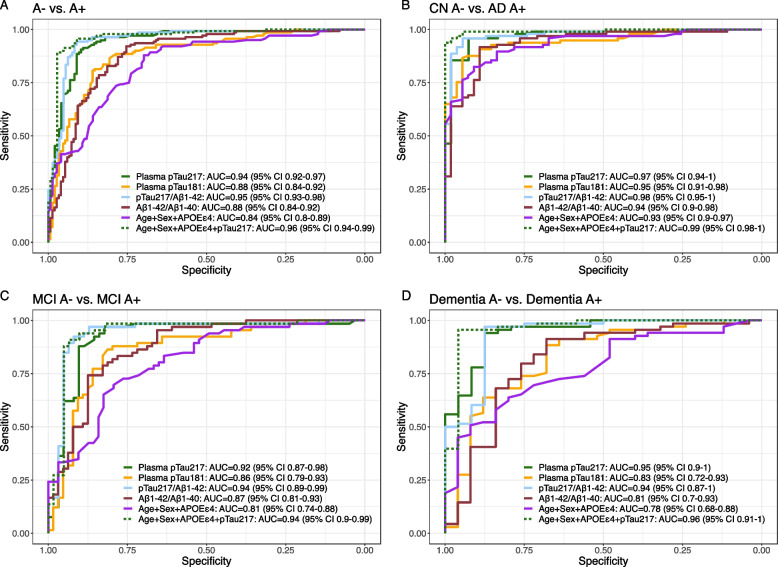
Diagnostic accuracy of plasma biomarkers for the discrimination of A + from A- categories. pTau_217_: phosphorylated tau 217, pTau_181_: phosphorylated tau 181. Aβ_1–42_: Amyloid β_1–42_. Aβ_1–40_: Amyloid β_1–40_. CN, cognitively unimpaired. MCI, mild cognitive impairment

In the whole sample, the AUC to discriminate A + from A- participants were 0.94 (95% Cl 0.92–0.97) for pTau_217_, and 0.88 (95% CI 0.84–0.92) for both pTau_181_ and Aβ_1–42_/Aβ_1–40_ (Fig. 3). The diagnostic accuracy of pTau_217_ to detect amyloid positivity was not outperformed by any other individual plasma biomarker, their ratios or their combinations. Aβ_1–42_ and Aβ_1–40_ individually had poor diagnostic accuracy, yielding AUCs below 0.70. Detailed two-by-two comparisons can be found as Supplementary Table 4. Sensitivity, specificity and Youden indices yielded by individual plasma markers are shown in Supplementary Fig. 7.

The diagnostic performance of pTau_217_ was also high across different clinical categories. In the MCI group, the accuracy of pTau_217_ (AUC = 0.92, 95% CI 0.87–0.98) was not significantly different than that pTau_181_ (AUC = 0.86, 95% CI 0.79–0.93) or that of the basic model with Age, Sex and *APOE ε4* (AUC = 0.81, 95% CI 0.74–0.88). However, the addition of pTau_217_ to Age, Sex and *APOE ε4* increased the accuracy of the model significantly from 0.81 (95% CI 0.74–0.88) to 0.94 (95% CI 0.9–0.99). pTau_217_ also showed very high accuracies (AUC = 0.97; 95% CI 0.94–1.00) to discriminate A + patients with a diagnosis of AD from different A- clinical groups (CN, other dementias and other not degenerative, Supplementary Fig. 8). The performance of plasma biomarkers to discriminate A + T + from A-T- participants yielded similar results (Supplementary Fig. 9).

### Cutoffs application

Table 2 shows the accuracy of different thresholds for pTau_217_, pTau_181_ and Aβ_1–42_/Aβ_1–40_ to detect amyloid positivity with a sensitivity and specificity of 97.5%, 95% and 90%. As pTau_217_ was the individual biomarker with highest diagnostic accuracy, we assessed the performance of selected cutoffs for this marker in different clinical groups, stratifying by decade. We found that the cutoff that had a global sensitivity of 95% (0.186 pg/mL) yielded accuracies above 84% across all decades and clinical groups when the amyloidosis prevalence was above 20%. In groups with lower amyloidosis prevalence (i.e. CN) the cutoff with specificity of 95% (0.388 pg/mL) showed higher accuracies. We observed that the cutoff that had a specificity of 95% showed a progressive decrease in accuracy with age in the MCI group (accuracy 84% in 60–69, 75% in 70–79 and 62% over 80 years). The negative predictive value of this cutoff was low over 70 years (58%). Detailed information on the accuracy of cutoffs in distinct clinical groups and decades can be found in Supplementary Material (tables 5, 6, 7 & 8).

**Table 2:** Thresholds for plasma pTau_217_, pTau_181_ and Aβ_1–42_/Aβ_1–40_ to detect A + participants

Threshold	Sensitivity	Specificity	NPV	PPV	Youden	Accuracy
Accuracy of plasma pTau_217_						
0.130	97.8%	65.5%	96.9%	73.1%	0.634	81.3%
0.186	95.0%	82.1%	94.4%	83.5%	0.770	88.4%
0.247	90.6%	89.7%	90.9%	89.4%	0.803	90.1%
0.249	89.9%	89.7%	90.3%	89.3%	0.796	89.8%
0.388	69.8%	95.2%	76.7%	93.3%	0.650	82.7%
0.552	45.3%	97.2%	65.0%	94.0%	0.426	71.8%
Accuracy of plasma pTau_181_						
1.573	97.9%	32.0%	94.1%	57.3%	0.299	63.8%
1.740	95.0%	44.7%	90.5%	61.6%	0.397	69.0%
2.125	90.0%	71.3%	88.4%	74.6%	0.613	80.3%
2.815	64.3%	90.0%	73.0%	85.7%	0.543	77.6%
3.345	47.9%	94.7%	66.0%	89.3%	0.425	72.1%
3.840	31.4%	97.3%	60.3%	91.7%	0.288	65.5%
Accuracy of Aβ_1–42_/Aβ_1–40_						
0.086	97.9%	41.3%	95.4%	60.9%	0.392	68.6%
0.080	95.0%	65.3%	93.3%	71.9%	0.603	79.7%
0.078	90.0%	75.3%	89.0%	77.3%	0.653	82.4%
0.073	65.0%	90.0%	73.4%	85.8%	0.550	77.9%
0.070	37.9%	94.7%	62.0%	86.9%	0.325	67.2%
0.067	20.7%	97.3%	56.8%	87.9%	0.180	60.3%

pTau_217_: phosphorylated tau 217, pTau_181_: phosphorylated tau 181. Aβ_1–42_: Amyloid β_1–42_. Aβ_1–40_: Amyloid β_1–40_. NPV, negative predictive value. PPV, positive predictive value. Threshold units for pTau are in pg/mL

We conducted a supervised decision tree analysis to determine the potential of various biomarkers and demographic factors in correctly identifying individuals with amyloidosis. This analysis incorporated plasma pTau_217_, pTau_181_, ratio Aβ_1–42_/ Aβ_1–40,_ Age, Sex, *APOE ε4* allele presence, and clinical diagnosis group (cognitively unimpaired, mild cognitive impairment, and dementia)*.* The most effective discriminators for amyloidosis were plasma pTau_217_ followed by the Aβ_1–42_/ Aβ_1–40_ ratio when pTau_217_ was high (Supplementary Fig. 10). Other variables were deemed less critical for amyloidosis detection and thus excluded from the decision tree. This algorithm exhibited a misclassification rate of 7.4%, with a sensitivity of 91%, specificity of 94%, overall accuracy of 93%, positive predictive value (PPV) of 94%, and negative predictive value (NPV) of 91%, accompanied by a false-negative rate (FNR) of 9.4% and a false-positive rate (FPR) of 5.6%.

### Potential of plasma pTau_217_ to predict Alzheimer disease pathophysiology

We assessed the predictive capability of pTau_217_ to properly classify participants with amyloidosis in our dataset, defined as CSF Aβ_1–42_/Aβ_1–40_ < 0.062. As the model that combined pTau_217_ with Age, Sex and *APOEε4* did not perform better than pTau_217_ alone in any comparison, we chose the simplest predictive model with pTau_217_. We bootstrapped to obtain robust predictive cuttoffs. We found no differences between the initial prediction and the mean of the 1000 predictive iterations, with a perfect correlation between them (Rho = 1, *p* < 0.001) thus reinforcing the robustness of our initial predictions.

**Fig. 4:**
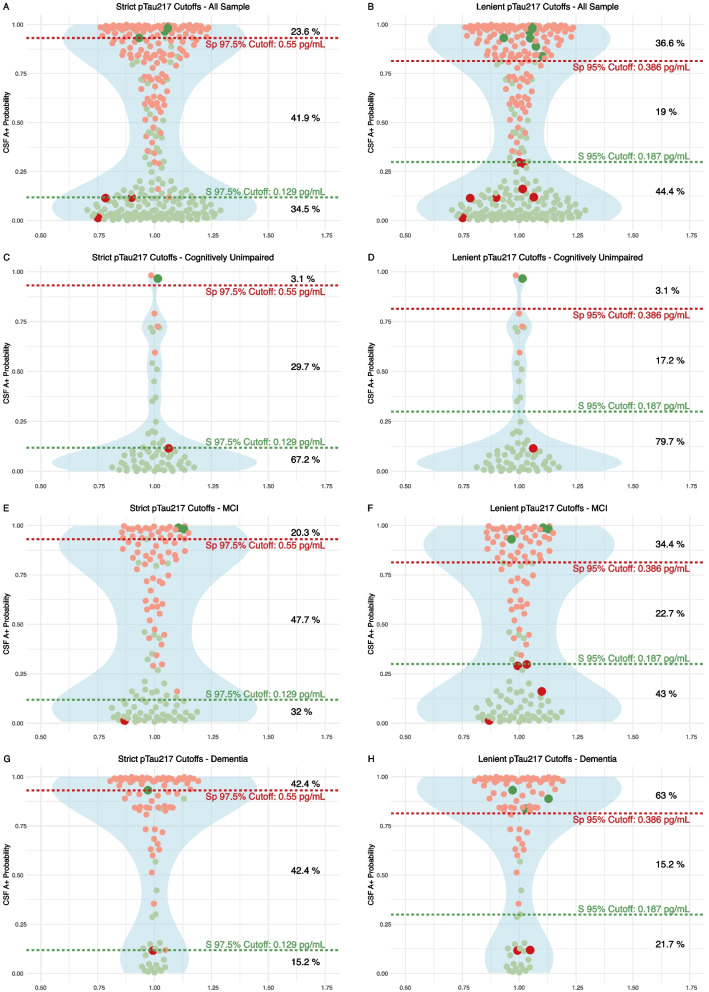
Plasma pTau217 predictive models. Strict and lenient cutoffs in the whole sample and in the distinct clinical groups. We illustrate the implementation of various cutoff thresholds within our sample, denoted by dashed lines in red and green at distinct Y-axis levels, each line representing the associated sensitivity and specificity values. Displayed in red dots are individuals with amyloid positivity in cerebrospinal fluid (CSF) and in green those amyloid negatives. Those participants with pTau_217_ concentrations above dashed red lines are classified as high risk of amyloid CSF positivity. The medium risk category is between dashed red and green lines. Below dashed green line are the participants classified as low risk. Observations that have been incorrectly categorized into high or low risk groups are represented by distinct sizes and colors. To the right, the corresponding percentages of the sample assigned to each risk category are presented. pTau_217_: phosphorylated tau 217, pTau_181_: phosphorylated tau 181. Aβ_1–42_: Amyloid β_1–42_. Aβ_1–40_: Amyloid β_1–40_. Model 1 included only plasma pTau_217_ for predictions. S, sensitivity. Sp, specificity

To facilitate the clinical implementation of plasma biomarkers while ensuring accuracy, we applied a two-threshold approach to classify participants into three groups, those with high, medium, and low likelihood of being CSF amyloid positive. Following this approach, those with a medium risk would benefit from a confirmation with gold standard tests like CSF or Amyloid PET. Figure 4 shows the thresholds in two different scenarios based on two levels of restrictiveness in sensitivity and specificity (97.5% and 95%). Using a highly accurate combination of cutoffs (one for 97.5% Sens and another for 97.5% Spec), only 41.9% of patients in the whole sample would require an additional test, with a global misclassification rate of 4.2%. Using less restrictive cutoffs (95% Sens/Spec), the proportion could be reduced to 19% with a global misclassification rate of 6%. Similar results were found in the subgroup of patients with CU, MCI and Dementia.

**Fig. 5:**
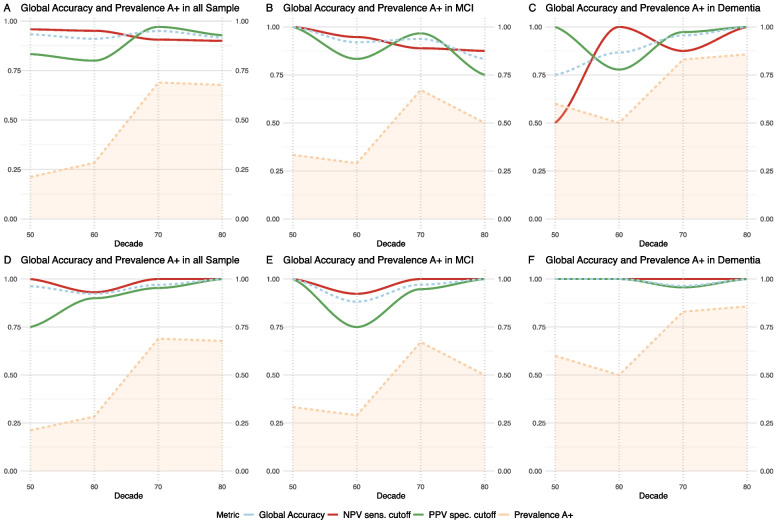
Negative predictive value, positive predictive value and global accuracy of pTau_217_ for the combination of cutoffs with 95% (A, B, C) and 97.5% (D, E, F) sensitivity and specificity and their relationship with age and prevalence of CSF Amyloidosis. The figure illustrates the association between the prevalence of cerebrospinal fluid (CSF) Amyloidosis (depicted by a dashed orange line), Positive Predictive Value (PPV, red line), Negative Predictive Value (NPV, green line), and global accuracy (dashed blue line) across different decades of life (X-axis) within our study cohort. In panels A-C, the PPV was determined using a cutoff for 95% specificity set at 0.386 pg/mL, while the NPV was ascertained using a 95% sensitivity cutoff at 0.187 pg/mL. In panels D-F, the PPV was determined using a cutoff for 97.5% specificity set at 0.55 pg/mL, while the NPV was ascertained using a 97.5% sensitivity cutoff at 0.129 pg/mL. The term 'global accuracy' in this context refers to the proportion of participants accurately classified as either positive or negative, based on the application of these two cutoffs. It should be noted that this calculation of global accuracy excludes participants categorized as 'indeterminate' (falling within the grey zone), for whom a confirmatory test is recommended. pTau_217_: phosphorylated tau 217, Sens, sensitivity. Spec, specificity. NPV, negative predictive value. PPV, positive predictive value. MCI, mild cognitive impairment

We finally assessed the robustness of those cutoffs in the whole sample, considering the increase of prevalence of amyloidosis with age in our population. In Fig. 5, we show the NPV, PPV, and global accuracy of pTau_217_ to detect A positivity by decades using cutoff combinations with 95% and 97.5% sensitivity and specificity in different clinical groups. We found high global accuracy (75%-100%) of the two-threshold application in all the scenarios, with variations of PPV and NPV according to CSF amyloid positive prevalence in our sample.

## Discussion

In this study, we found that the concentration of plasma pTau_217_, plasma pTau_181_, and the ratio Aβ_1–42_/Aβ_1–40_ measured in a fully automated platform, yielded excellent accuracy to detect the AD pathophysiology in the setting of the routine clinical practice of a memory clinic. Of all plasma markers, the recently developed assay that measures pTau_217_ was the most accurate followed by the Aβ_1–42_/Aβ_1–40_ ratio and pTau_181_. We also found that different comorbidities had a mild significant effect on plasma markers of AD, but the amyloid status was the single variable with the largest effect on their concentration. In patients with advanced chronic kidney disease, the use of ratios could reduce the impact of having higher plasma concentrations associated to low renal function. Furthermore, we applied predictive models to obtain stratification profiles that showed the potential to reduce the need of more costly or invasive procedures by approximately 60 to 80%.

The performance of plasma markers to detect the AD pathophysiology has been assessed in previous studies using different analytical platforms, with AUCs ranging from 0.70 to 0.96 for pTau_181_ [7, 23, 39–43], and from 0.64 to 0.86 for Aβ_1–42_/Aβ_1–40_ [5, 8, 10]. Plasma pTau_231_ and pTau_217_ have shown to better capture the earliest cerebral Aβ changes in CU, before overt Aβ plaque pathology is present [26] For plasma pTau_217_, accuracies have varied depending on the platform, yet the performance has consistently been high in discriminating amyloidosis in MCI and predicting progression, typically outperforming that of other plasma pTau isoforms [7, 23, 33, 44]. Most research studies reported better accuracies with the use of composite measures that combined two or more markers and/or clinical or genetic information [45–47]. In our study, plasma pTau_217,_ the Aβ_1–42_/Aβ_1–40_ ratio and pTau_181_ measured with a fully automated platform showed high diagnostic performance to detect amyloid positivity. Of these, pTau_217_ was the marker that showed higher accuracy and, importantly, it was not outperformed by composite measures indicating that it is a good candidate for its implementation as a single biomarker.

Automated platforms have revolutionized CSF analysis by resolving critical analytical factors and, similarly, they hold promise for transforming plasma testing, enhancing both precision and efficiency in biomarker quantification. Recent studies have assessed the diagnostic performance of pTau_181_ and Aβ_1–42_/Aβ_1–40_ in the Lumipulse platform. Janelidze et al. reported an AUC of 0.7 for pTau_181_ for the identification of CSF amyloidosis in MCI [23]and of 0.74 to detect progression to dementia. However, Wilson et al. reported a higher accuracy of 0.96 [40] for the discrimination between Aβ- CU and Aβ + AD patients. Another recent study using the Lumipulse platform analyzed the accuracy to detect AD of plasma pTau_181_ and Aβ_1–42_/Aβ_1–40_ ratio in cognitively unimpaired participants and found that Aβ_1–42_/Aβ_1–40_ ratio was the most cost-effective (AUC 0.9 for A + and 0.89 for A + T +), followed by pTau_181_ that showed an AUC of 0.76 for A + and 0.86 for A + T + [48]. Our results are in line with previous studies, and show a global accuracy of 0.88 for plasma pTau_181_ and for the plasma Aβ_1–42_/Aβ_1–40_ ratio. A variety of reasons could explain the minor discrepancies between studies, including differences in preanalytical conditions [49], in the kits that were used, characteristics of the sample and cohorts, and the design of the studies. Our design including consecutive patients that underwent a lumbar puncture for routine diagnostic work-up in a memory clinic setting, provides information about the potential implementation of plasma markers in this context.

Together with the commercially available pTau_181_ and Aβ_1–42_/Aβ_1–40_, we evaluated the performance of plasma pTau_217_, recently developed for the same automated platform, and found that it outperformed pTau_181_ and Aβ_1–42_/Aβ_1–40_ in the detection of amyloid positivity. In previous research studies, plasma pTau_217_ has consistently shown exceptionally high accuracy across different platforms and has demonstrated strong correlations with other markers of AD (CSF biomarkers, amyloid PET and Tau PET) and with neuropathology [30]. The fact that it has shown greatest fold changes and effect sizes compared to other pTau isoforms, makes it a perfect candidate for its implementation in clinical settings, as small analytical variations (5–10%) would not substantially affect its diagnostic performance. Its implementation on a fully automated platform would not only simplify the process but also enhance accessibility for clinical laboratories. In our study using a fully automated platform, we found that A + patients had 4.42 times higher concentrations of plasma pTau_217_. This magnitude of effect, combined with the advantages of automation, makes this assay particularly promising for integration into standard clinical practices.

To facilitate a more rational utilization of plasma biomarkers, we followed the methodology delineated by Brum et al. stratifying the risk of having AD pathopshysiology [38]. By implementing this strategy, it is feasible to define threshold values that are highly sensitive and specific to either detect or rule out CSF amyloidosis minimizing the probability of misclassification. This would also allow for selecting those patients that fall within an intermediate likelihood category or ‘grey zone’ and that would benefit of further etiological investigations (CSF biomarkers, amyloid or Tau PET). This stratification framework represents a pragmatic and flexible approach for the incorporation of plasma biomarkers in clinical settings. An open point of discussion is the optimal context of use —primary care, general neurology, or specialized memory clinics— and the management strategies for participants identified as high or low risk. Our study showed that following this approach, pTau_217_ had an excellent performance in the context of a specialized memory clinic, but these classifications will need to be contextualized within other clinical settings [4, 34]. The effect of comorbidities as CKD on plasma biomarker concentrations points in the same direction as recently published studies [15, 50], in which the use of ratios could attenuate the effect of CKD. Moreover, we found that the effect of renal dysfunction on plasma pTau_217_ concentrations was significantly less than that of the amyloid positivity status, suggesting that the actual impact of CKD on the diagnostic performance of this marker would be minimal. When we assessed the impact of renal dysfunction on different plasma pTau biomarker concentrations, we found that the effect size of eGFR < 60 mL/min/1.73m3 in pTau_181_ was double that observed in pTau_217_, with standardized beta coefficients of 0.25 and 0.11, respectively (Fig. 2), both statistically significant. When we compared the AUC of plasma pTau_217_ and pTau_181_ in the subset of patients with eGFR < 60 mL/min/1.73m3, although higher in pTau_217_ (AUC 0.83, CI 95% 0.64–1) than in pTau_181_ (AUC 0.75, CI 95% 0.53–0.97), accuracy did not differ between both biomarkers, probably because of low statistical power to capture differences in this subset in our cohort, as only 11 patients were A- and 12 patients A + .

One of the strengths of our study is that we included all consecutive participants from routine clinical practice that underwent lumbar puncture throughout one year in our memory clinic including a variety of diagnoses. This approach reduces the risk of selection biases and ensures a reliable representation of the population assessed in the setting of a specialized memory clinic, also providing relevant information on their potential implementation in the routine diagnostic work-up in this context. Other strengths in our study are the fact that all markers were measured using the same batch of reagents and that the clinical information available allowed us to analyze the potential impact of comorbidities and perform sub analyses within distinct clinical stages.

## Limitations

Our study also has some limitations. First, as the inclusion criteria required that participants had received a lumbar puncture for CSF biomarkers, the extrapolation to other contexts of use different than specialized memory units, such as primary care or population screening programs, should be made cautiously. Second, we could not compare plasma pTau_217_ with its counterpart in CSF as the assay was specifically developed for plasma. Another limitation is the lack of Amyloid/Tau PET or neuropathological confirmation in our participants, and although the CSF biomarker cutoffs in our center were validated against amyloid PET [37], we cannot be certain about how using a different gold-standard might affect our results. Finally, this is a single-center study, and even though this increases uniformity and we performed a bootstrapped cross-validation to ensure the predictive robustness of our models, the accuracy and true predictive power of the cutoffs derived from our results need to be verified in diverse datasets with comparable characteristics.

## Conclusions

Our study provides evidence that plasma markers can reliably be measured in an automated platform, and highlights plasma pTau_217_ as the most promising plasma biomarker, showing great potential for the detection of AD pathophysiology in the context of a memory clinic. With the arrival of disease-modifying treatments into clinical practice, it is urgent to have easily accessible and efficient diagnostic methods to identify patients that could benefit from these therapies. The implementation of plasma biomarkers in readily accessible fully automated platforms will streamline the diagnosis and enhance the accessibility of disease modifying therapies.

### Supplementary Information

Supplementary Material 1.

## Data Availability

Raw anonymized data and code for statistical analysis are available upon reasonable request. All requests should be sent to the corresponding author detailing the study hypothesis and statistical analysis plan. The steering committee of this study will decide whether data/code sharing is appropriate based on the novelty and scientific rigor of the proposal. All applicants will be asked to sign a data access agreement.

## References

[1] Fargo KN, Carrillo MC, Weiner MW, et al. The crisis in recruitment for clinical trials in Alzheimer’s and dementia: An action plan for solutions. Alzheimer’s and Dementia. 2016;12:1113–5. 10.1016/j.jalz.2016.10.001. 27836052 10.1016/j.jalz.2016.10.001

[2] Teunissen CE, Verberk IMW, Thijssen EH, et al. Blood-based biomarkers for Alzheimer’s disease: towards clinical implementation. Lancet Neurol. 2022;21:66–77. 10.1016/S1474-4422(21)00361-6. 34838239 10.1016/S1474-4422(21)00361-6

[3] Alcolea D, Delaby C, Muñoz L, et al. Use of plasma biomarkers for AT(N) classification of neurodegenerative dementias. J Neurol Neurosurg Psychiatry. 2021;92:1206–14. 34103344 10.1136/jnnp-2021-326603

[4] Alcolea D, Beeri MS, Rojas JC, et al. Blood Biomarkers in Neurodegenerative Diseases: Implications for the Clinical Neurologist. Neurology. 2023. 10.1212/WNL.0000000000207193. 36878698 10.1212/WNL.0000000000207193 PMC10435056

[5] Nakamura A, Kaneko N, Villemagne VL, et al. High performance plasma amyloid-β biomarkers for Alzheimer’s disease. Nature. 2018;554:249–54. 29420472 10.1038/nature25456

[6] Janelidze S, Stomrud E, Palmqvist S, et al. Plasma β-amyloid in Alzheimer’s disease and vascular disease. Sci Rep. 2016;6. 10.1038/srep26801. 10.1038/srep26801 PMC4886210 27241045

[7] Ashton NJ, Puig-Pijoan A, Milà-Alomà M, et al. Plasma and CSF biomarkers in a memory clinic: Head-to-head comparison of phosphorylated tau immunoassays. Alzheimer’s Dement Published Online First. 2022. 10.1002/alz.12841. 10.1002/alz.12841 PMC10762642 36370462

[8] Schindler SE, Bollinger JG, Ovod V, et al. High-precision plasma β-amyloid 42/40 predicts current and future brain amyloidosis. Neurology. 2019;93:E1647–59. 31371569 10.1212/WNL.0000000000008081 PMC6946467

[9] Montoliu-Gaya L, Strydom A, Blennow K, et al. Blood biomarkers for alzheimer’s disease in down syndrome. J Clin Med. 2021;10. 10.3390/jcm10163639. 10.3390/jcm10163639 PMC8397053 34441934

[10] Janelidze S, Teunissen CE, Zetterberg H, et al. Head-to-Head Comparison of 8 Plasma Amyloid-β 42/40 Assays in Alzheimer Disease. JAMA Neurol. 2021;78:1375–82. 34542571 10.1001/jamaneurol.2021.3180 PMC8453354

[11] Bayoumy S, Verberk IMW, den Dulk B, et al. Clinical and analytical comparison of six Simoa assays for plasma P-tau isoforms P-tau181, P-tau217, and P-tau231. Alzheimers Res Ther. 2021;13. 10.1186/s13195-021-00939-9. 10.1186/s13195-021-00939-9 PMC8645090 34863295

[12] Illán-Gala I, Lleo A, Karydas A, et al. Plasma Tau and Neurofilament Light in Frontotemporal Lobar Degeneration and Alzheimer Disease. Neurology. 2021;96:e671–83. 33199433 10.1212/WNL.0000000000011226 PMC7884995

[13] Mielke MM, Dage JL, Frank RD, et al. Performance of plasma phosphorylated tau 181 and 217 in the community. Nat Med. 2022;28:1398–405. 35618838 10.1038/s41591-022-01822-2 PMC9329262

[14] Pichet Binette A, Janelidze S, Cullen N, et al. Confounding factors of Alzheimer’s disease plasma biomarkers and their impact on clinical performance. Alzheimer’s and Dementia Published Online First. 2022. 10.1002/alz.12787. 10.1002/alz.12787 PMC10499000 36152307

[15] Syrjanen JA, Campbell MR, Algeciras-Schimnich A, et al. Associations of amyloid and neurodegeneration plasma biomarkers with comorbidities. Alzheimer’s Dement. 2022;18:1128–40. 34569696 10.1002/alz.12466 PMC8957642

[16] Ashton NJ, Janelidze S, Mattsson-Carlgren N, et al. Differential roles of Aβ42/40, p-tau231 and p-tau217 for Alzheimer’s trial selection and disease monitoring. Nat Med. 2022;28:2555–62. 36456833 10.1038/s41591-022-02074-w PMC9800279

[17] Verberk IMW, Slot RE, Verfaillie SCJ, et al. Plasma Amyloid as Prescreener for the Earliest Alzheimer Pathological Changes. Ann Neurol. 2018;84:648–58. 30196548 10.1002/ana.25334 PMC6282982

[18] Pichet Binette A, Palmqvist S, Bali D, et al. Combining plasma phospho-tau and accessible measures to evaluate progression to Alzheimer’s dementia in mild cognitive impairment patients. Alzheimers Res Ther. 2022;14. 10.1186/s13195-022-00990-0. 10.1186/s13195-022-00990-0 PMC8966264 35351181

[19] Palmqvist S, Tideman P, Cullen N, et al. Prediction of future Alzheimer’s disease dementia using plasma phospho-tau combined with other accessible measures. Nat Med Published Online First. 2021. 10.1038/s41591-021-01348-z. 10.1038/s41591-021-01348-z 34031605

[20] Mattsson-Carlgren N, Salvadó G, Ashton NJ, et al. Prediction of Longitudinal Cognitive Decline in Preclinical Alzheimer Disease Using Plasma Biomarkers. JAMA Neurol. 2023;80:360–9. 36745413 10.1001/jamaneurol.2022.5272 PMC10087054

[21] Teunissen CE, Thijssen EH. Plasma p-tau217: From ‘new kid’ to most promising candidate for Alzheimer’s disease blood test. Brain. 2020;143:3170–80. 10.1093/BRAIN/AWAA329. 33278818 10.1093/BRAIN/AWAA329 PMC7719020

[22] Salvadó G, Ossenkoppele R, Ashton NJ, et al. Specific associations between plasma biomarkers and postmortem amyloid plaque and tau tangle loads. EMBO Mol Med. 2023;15. 10.15252/emmm.202217123. 10.15252/emmm.202217123 PMC10165361 36912178

[23] Janelidze S, Bali D, Ashton NJ, et al. Head-to-head comparison of 10 plasma phospho-tau assays in prodromal Alzheimer’s disease. Brain. Published Online First. 2022. 10.1093/brain/awac333. 10.1093/brain/awac333 PMC10115176 36087307

[24] Ashton NJ, Brum WS, Di Molfetta G, et al. Diagnostic accuracy of the plasma ALZpath pTau217 immunoassay to identify Alzheimer’s disease pathology. medRxiv. Published Online First: 12 July 2023. 10.1101/2023.07.11.23292493.

[25] Cullen NC, Janelidze S, Mattsson-Carlgren N, et al. Test-retest variability of plasma biomarkers in Alzheimer’s disease and its effects on clinical prediction models. Alzheimer’s Dement. 2023;19:797–806. 10.1002/alz.12706 PMC9747985 35699240

[26] Milà-Alomà M, Ashton NJ, Shekari M, et al. Plasma p-tau231 and p-tau217 as state markers of amyloid-β pathology in preclinical Alzheimer’s disease. Nat Med. 2022;28:1797–801. 35953717 10.1038/s41591-022-01925-w PMC9499867

[27] Yu L, Boyle PA, Janelidze S, et al. Plasma p-tau181 and p-tau217 in discriminating PART, AD and other key neuropathologies in older adults. Acta Neuropathol. 2023;146:1–11. 37031430 10.1007/s00401-023-02570-4 PMC10261204

[28] Pais MV, Forlenza OV, Diniz BS. Plasma Biomarkers of Alzheimer’s Disease: A Review of Available Assays, Recent Developments, and Implications for Clinical Practice. J Alzheimers Dis Rep. 2023;7:355–80. 37220625 10.3233/ADR-230029 PMC10200198

[29] Jack CR, Wiste HJ, Algeciras-Schimnich A, et al. Predicting amyloid PET and tau PET stages with plasma biomarkers. Brain. 2023;146:2029–44. 36789483 10.1093/brain/awad042 PMC10151195

[30] Therriault J, Vermeiren M, Servaes S, et al. Association of Phosphorylated Tau Biomarkers with Amyloid Positron Emission Tomography vs Tau Positron Emission Tomography. JAMA Neurol. 2023;80:188–99. 36508198 10.1001/jamaneurol.2022.4485 PMC9856704

[31] Therriault J, Servaes S, Tissot C, et al. Equivalence of plasma p-tau217 with cerebrospinal fluid in the diagnosis of Alzheimer’s disease. Alzheimer’s Dement Published Online First. 2023. 10.1002/alz.13026. 10.1002/alz.13026 PMC10587362 37078495

[32] Groot C, Cicognola C, Bali D, et al. Diagnostic and prognostic performance to detect Alzheimer’s disease and clinical progression of a novel assay for plasma p-tau217. Alzheimers Res Ther. 2022;14. 10.1186/s13195-022-01005-8. 10.1186/s13195-022-01005-8 PMC9107269 35568889

[33] Palmqvist S, Janelidze S, Quiroz YT, et al. Discriminative Accuracy of Plasma Phospho-tau217 for Alzheimer Disease vs Other Neurodegenerative Disorders. J Am M Assoc. 2020;324:772–81. 10.1001/jama.2020.12134 PMC7388060 32722745

[34] Hansson O, Edelmayer RM, Boxer AL, et al. The Alzheimer’s Association appropriate use recommendations for blood biomarkers in Alzheimer’s disease. Alzheimer’s Dement. 2022;18:2669–86. 10.1002/alz.12756. 35908251 10.1002/alz.12756 PMC10087669

[35] Alcolea D, Clarimón J, Carmona-Iragui M, et al. The Sant Pau Initiative on Neurodegeneration (SPIN) cohort: a data set for biomarker discovery and validation in neurodegenerative disorders. Alzheimer’s Dement. 2019;5:597–609. 10.1016/j.trci.2019.09.005 PMC6804606 31650016

[36] Mansilla A, Canyelles M, Ferrer R, et al. Effects of storage conditions on the stability of blood-based markers for the diagnosis of Alzheimer’s disease. Clin Chem Lab Med. 2023;61:1580–9. 37083158 10.1515/cclm-2023-0245

[37] Alcolea D, Pegueroles J, Muñoz L, et al. Agreement of amyloid PET and CSF biomarkers for Alzheimer’s disease on Lumipulse. Ann Clin Transl Neurol. 2019;6:1815–24. 31464088 10.1002/acn3.50873 PMC6764494

[38] Brum WS, Cullen NC, Janelidze S, et al. A two-step workflow based on plasma p-tau217 to screen for amyloid β positivity with further confirmatory testing only in uncertain cases. Nat Aging Published Online First. 2023. 10.1038/s43587-023-00471-5. 10.1038/s43587-023-00471-5 PMC10501903 37653254

[39] Baiardi S, Quadalti C, Mammana A, et al. Diagnostic value of plasma p-tau181, NfL, and GFAP in a clinical setting cohort of prevalent neurodegenerative dementias. Alzheimers Res Ther. 2022;14. 10.1186/s13195-022-01093-6. 10.1186/s13195-022-01093-6 PMC9555092 36221099

[40] Wilson EN, Young CB, Ramos Benitez J, et al. Performance of a fully-automated Lumipulse plasma phospho-tau181 assay for Alzheimer’s disease. Alzheimers Res Ther. 2022;14. 10.1186/s13195-022-01116-2. 10.1186/s13195-022-01116-2 PMC9652927 36371232

[41] Thijssen EH, La Joie R, Strom A, et al. Plasma phosphorylated tau 217 and phosphorylated tau 181 as biomarkers in Alzheimer’s disease and frontotemporal lobar degeneration: a retrospective diagnostic performance study. Lancet Neurol. 2021;20:739–52. 34418401 10.1016/S1474-4422(21)00214-3 PMC8711249

[42] Karikari TK, Pascoal TA, Ashton NJ, et al. Blood phosphorylated tau 181 as a biomarker for Alzheimer’s disease: a diagnostic performance and prediction modelling study using data from four prospective cohorts. Lancet Neurol. 2020;19:422–33. 32333900 10.1016/S1474-4422(20)30071-5

[43] Sarto J, Ruiz-García R, Guillén N, et al. Diagnostic Performance and Clinical Applicability of Blood-Based Biomarkers in a Prospective Memory Clinic Cohort. Neurology. 2022. 10.1212/WNL.0000000000201597. 36450604 10.1212/WNL.0000000000201597 PMC9984216

[44] Brickman AM, Manly JJ, Honig LS, et al. Plasma p-tau181, p-tau217, and other blood-based Alzheimer’s disease biomarkers in a multi-ethnic, community study. Alzheimer’s and Dementia. 2021;17:1353–64. 33580742 10.1002/alz.12301 PMC8451860

[45] Cullen NC, Leuzy A, Palmqvist S, et al. Individualized prognosis of cognitive decline and dementia in mild cognitive impairment based on plasma biomarker combinations. Nat Aging. 2021;1:114–23. 37117993 10.1038/s43587-020-00003-5

[46] Janelidze S, Palmqvist S, Leuzy A, et al. Detecting amyloid positivity in early Alzheimer’s disease using combinations of plasma Aβ42/Aβ40 and p-tau. Alzheimer’s Dement. 2022;18:283–93. 34151519 10.1002/alz.12395

[47] Palmqvist S, Stomrud E, Cullen N, et al. An accurate fully automated panel of plasma biomarkers for Alzheimer’s disease. Alzheimer’s Dement. 2023;19:1204–15. 35950735 10.1002/alz.12751 PMC9918613

[48] Martínez-Dubarbie F, Guerra-Ruiz A, López-García S, et al. Accuracy of plasma Aβ40, Aβ42, and p-tau181 to detect CSF Alzheimer’s pathological changes in cognitively unimpaired subjects using the Lumipulse automated platform. Alzheimers Res Ther. 2023;15:163. 37784138 10.1186/s13195-023-01319-1 PMC10544460

[49] Musso G, Cosma C, Zaninotto M, et al. Pre-analytical variability of the Lumipulse immunoassay for plasma biomarkers of Alzheimer’s disease. Clin Chem Lab Med. 2023;61:E53–6. 10.1515/cclm-2022-0770. 36423341 10.1515/cclm-2022-0770

[50] Janelidze S, Barthélemy NR, He Y, et al. Mitigating the Associations of Kidney Dysfunction With Blood Biomarkers of Alzheimer Disease by Using Phosphorylated Tau to Total Tau Ratios. JAMA Neurol. Published Online First. 2023. 10.1001/jamaneurol.2023.0199. 10.1001/jamaneurol.2023.0199 PMC10061310 36987840

