# Correction: Diagnostic performance of plasma pTau_217_, pTau_181_, Aβ_1‑42_ and Aβ_1‑40_ in the LUMIPULSE automated platform for the detection of Alzheimer disease

**DOI:** 10.1186/s13195-024-01538-0

**Published:** 2024-07-27

**Authors:** Javier Arranz, Nuole Zhu, Sara Rubio-Guerra, Íñigo Rodríguez-Baz, Rosa Ferrer, María Carmona-Iragui, Isabel Barroeta, Ignacio Illán-Gala, Miguel Santos-Santos, Juan Fortea, Alberto Lleó, Mireia Tondo, Daniel Alcolea

**Affiliations:** 1 Sant Pau Memory Unit, Department of Neurology, IR SANT PAU, Hospital de La Santa Creu I Sant Pau, C/Sant Quintí 89, 08041 Barcelona, Spain; 2 Department of Neurology, Unidad Alzheimer-Down, IR SANT PAU, Hospital de La Santa Creu I Sant Pau; Barcelona Down Medical Center, Fundació Catalana Síndrome de Down, Barcelona, Spain; 3 Universitat Autònoma de Barcelona, Barcelona, Spain; 4 Centro de Investigación Biomédica en Red en Enfermedades Neurodegenerativas, CIBERNED, Madrid, Spain; 5 Servei de Bioquímica I Biologia Molecular, IR SANT PAU, Hospital de La Santa Creu I Sant Pau, Universitat Autònoma de Barcelona, C/Sant Quintí 89, 08041 Barcelona, Spain; 6 Centro de Investigación Biomédica en Red en Diabetes y Enfermedades Metabólicas, CIBERDEM, Madrid, Spain

**Correction:** ***Alz Res Therapy*** **16, 139 (2024)**

10.1186/s13195-024-01513-9.

Following publication of the original article [1], the authors corrected the Funding section to mention “project PI22/00611”.

The original article [1] has been corrected.
