# Supplementary material for: Diagnostic performance of plasma pTau217, pTau181, Aβ1-42 and Aβ1-40 in the LUMIPULSE automated platform for the detection of Alzheimer disease
Source: Alzheimers Res Ther. 2024 Jun 26;16:139. doi: 10.1186/s13195-024-01513-9 (PMC11200993; doi:10.1186/s13195-024-01513-9)

**SUPPLEMENTARY MATERIAL**

**Supplementary Text 1: Cognitively unimpaired participants in the SPIN cohort.**

**Asymptomatic volunteers are an extremely relevant group in the SPIN cohort. Volunteers are usually spouses or children of patients that are informed about our studies at the outpatient clinics of the Sant Pau Memory Unit. Volunteers can also learn about our projects through talks, our website (https://santpaumemoryunit.com) or social media (@SantPauMemory). All volunteers receive an initial consultation with a neurologist in which the SPIN protocol is explained in detail, inclusion/exclusion criteria are reviewed and informed consent is signed. More information about the evaluation protocol in cognitively normal participants of the SPIN cohort can be found in Alcolea et al. Alzheimer’s Dement (NY) 2019. Five out of the 66 cognitively unimpaired participants included in this study had subjective cognitive decline but full neuropsychological tests within normal range.**

**Supplementary Table 1: Demographics according to clinical diagnosis**

Unless otherwise specified, quantitative measures are presented as mean (SD).


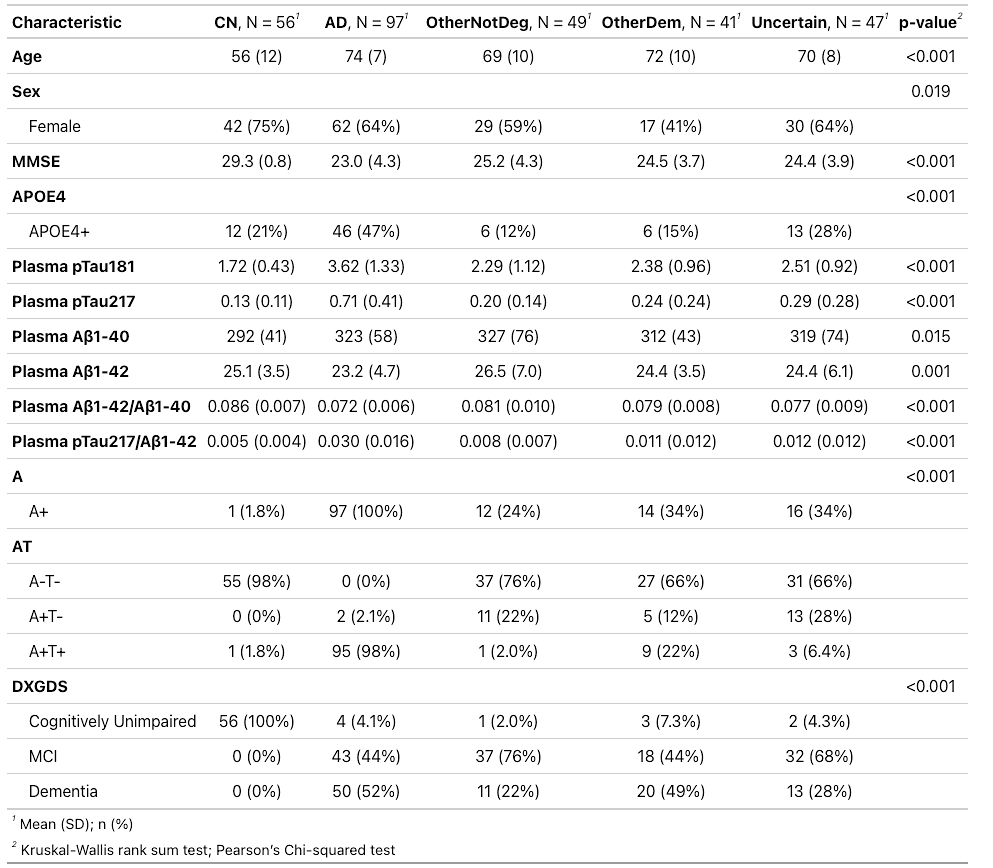
pTau_217_, phosphorylated tau 217, pTau_181_, phosphorylated tau 181. Aβ_1–42_, Amyloid β_1–42_. Aβ_1–40_, Amyloid β_1–40_. CU, cognitively unimpaired. OtherDem, other dementias. OtherNotDeg, not degenerative dementias.

Kruskal-Wallis rank sum test was used to compare continuous variables that were not normally distributed; Pearson’s Chi-squared test was used to compare categorical variables.

*Cognitively unimpaired etiologies: cognitively normal/healthy controls (n=56), other not neurodegenerative (n=1), preclinical AD stage 1 (n=2), preclinical AD stage 2 (n=2), Uncertain etiology (n=2), Vascular (n=3).

**Supplementary Table 2: Demographics according to A status**

Unless otherwise specified, quantitative measures are presented as mean (SD).

pTau_217_, phosphorylated tau 217, pTau_181_, phosphorylated tau 181. Aβ_1–42_, Amyloid β_1–42_. Aβ_1–40_, Amyloid β_1–40_. CU, cognitively unimpaired. OtherDem, other dementias. OtherNotDeg, not degenerative dementias.


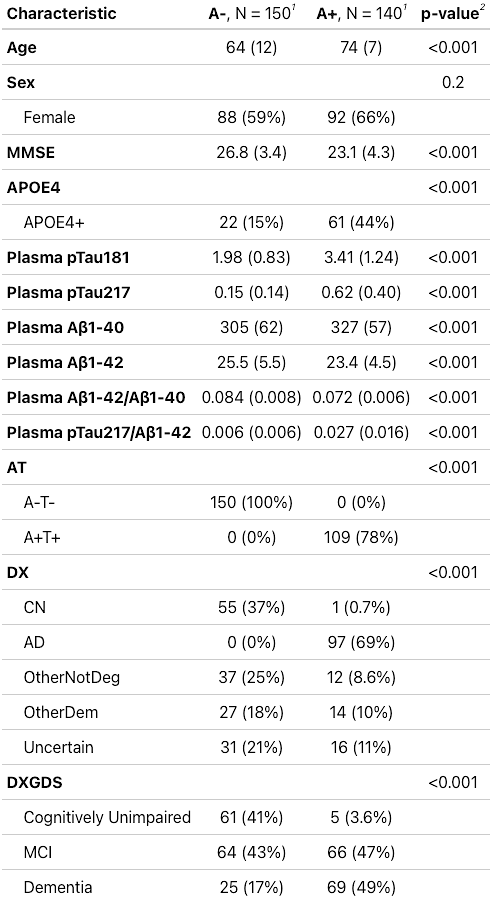

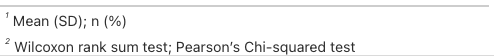


Wilcoxon rank sum test was used to compare continuous variables that were not normally distributed; Pearson’s Chi-squared test was used to compare categorical variables.

**Supplementary Table 3: Inter-assay coefficients of variations of plasma biomarkers measured with the LUMIPULSE platform.**

| **Analyte** | **Low Concentration CV (%)** | **Low Concentration (pg/mL)** | **High Concentration CV (%)** | **High Concentration (pg/mL)** |
| --- | --- | --- | --- | --- |
| pTau_217_ | 4.5 | 0.58 | 5.7 | 4.59 |
| pTau_181_ | 4.6 | 5 | 4.1 | 45 |
| Aβ_1–42_ | 5.7 | 21 | 6.6 | 209 |
| Aβ_1–40_ | 5.5 | 214 | 6.7 | 2243 |

pTau_217_, phosphorylated tau 217, pTau_181_, phosphorylated tau 181. Aβ_1–42_, Amyloid β_1–42_. Aβ_1–40_, Amyloid β_1–40_. CV, coefficient of variation.

**Supplementary Table 4: Comparison of plasma biomarkers’ accuracy using DeLong test adjusted by multiple comparisons**

| **Contrast** | **Biomarker 1** | **AUC1** | **Biomarker 2** | **AUC2** | **P value (unadjusted)** | **P value (Bonferroni-adjusted)** |
| --- | --- | --- | --- | --- | --- | --- |
| A- vs. A+ | pTau_217_ | 0.94 | pTau_181_ | 0.88 | 4.5111E-05 | <0.05 (comp.mult) |
| A- vs. A+ | pTau_217_ | 0.94 | pTau_217_/Aβ_1–42_ | 0.95 | 0.0434699 | ns |
| A- vs. A+ | pTau_217_ | 0.94 | Aβ_1–42_/Aβ_1–40_ | 0.88 | 0.008582 | ns |
| A- vs. A+ | pTau_217_ | 0.94 | Age+Sex+ *APOEε*4 | 0.84 | 5.9871E-05 | <0.05 (comp.mult) |
| A- vs. A+ | pTau_217_ | 0.94 | Age+Sex+ *APOEε*4+pTau_217_ | 0.96 | 0.00468322 | ns |
| A- vs. A+ | pTau_181_ | 0.88 | pTau_217_/Aβ_1–42_ | 0.95 | 9.3803E-06 | <0.05 (comp.mult) |
| A- vs. A+ | pTau_181_ | 0.88 | Aβ_1–42_/Aβ_1–40_ | 0.88 | 0.99792624 | ns |
| A- vs. A+ | pTau_181_ | 0.88 | Age+Sex+ *APOEε*4 | 0.84 | 0.16609191 | ns |
| A- vs. A+ | pTau_181_ | 0.88 | Age+Sex+ *APOEε*4+pTau_217_ | 0.96 | 1.3124E-06 | <0.05 (comp.mult) |
| A- vs. A+ | pTau_217_/Aβ_1–42_ | 0.95 | Aβ_1–42_/Aβ_1–40_ | 0.88 | 0.00197812 | <0.05 (comp.mult) |
| A- vs. A+ | pTau_217_/Aβ_1–42_ | 0.95 | Age+Sex+ *APOEε*4 | 0.84 | 1.0157E-05 | <0.05 (comp.mult) |
| A- vs. A+ | pTau_217_/Aβ_1–42_ | 0.95 | Age+Sex+ *APOEε*4+pTau_217_ | 0.96 | 0.09589594 | ns |
| A- vs. A+ | Aβ_1–42_/Aβ_1–40_ | 0.88 | Age+Sex+ *APOEε*4 | 0.84 | 0.14280983 | ns |
| A- vs. A+ | Aβ_1–42_/Aβ_1–40_ | 0.88 | Age+Sex+ *APOEε*4+pTau_217_ | 0.96 | 0.00023526 | <0.05 (comp.mult) |
| A- vs. A+ | Age+Sex+*APOEε*4 | 0.84 | Age+Sex+ *APOEε*4+pTau_217_ | 0.96 | 2.3492E-08 | <0.05 (comp.mult) |

AD plasma biomarkers and their combinations that better discriminate A+ from A-. We included those with AUC >0.80 and p<0.05. pTau_217_, phosphorylated tau 217, pTau_181_, phosphorylated tau 181. Aβ_1–42_, Amyloid β_1–42_. Aβ_1–40_, Amyloid β_1–40_.

**Supplementary Table 5: Plasma pTau217 accuracy to detect A positivity stratifying by cognitive status and age using the cutoff with a fixed sensititivy of 95%**

MCI, mild cognitive impairment. S, sensitivity; Sp, specificity; NPV, negative predictive value; PPV, positive predictive value. pTau_217_, phosphorylated tau 217.

The small number of participants with age under 60 and above 80 might alter accuracy performance for these age ranges due to potential overfitting of cutoffs in these subgroups.


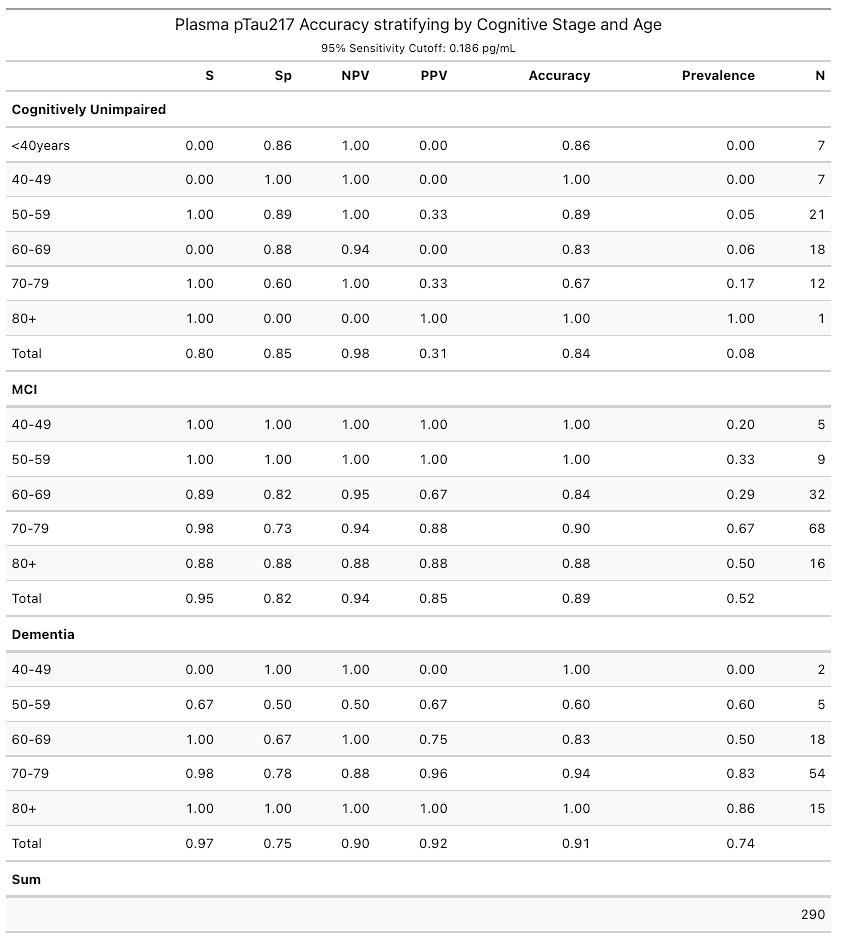


**Supplementary Table 6: Plasma pTau217 accuracy to detect A positivity stratifying by cognitive status and age using a cutoff with a fixed specificity of 95%**

MCI, mild cognitive impairment. S, sensitivity; Sp, specificity; NPV, negative predictive value; PPV, positive predictive value. pTau_217_, phosphorylated tau 217.

The small number of participants with age under 60 and above 80 might alter accuracy performance for these age ranges due to potential overfitting of cutoffs in these subgroups.


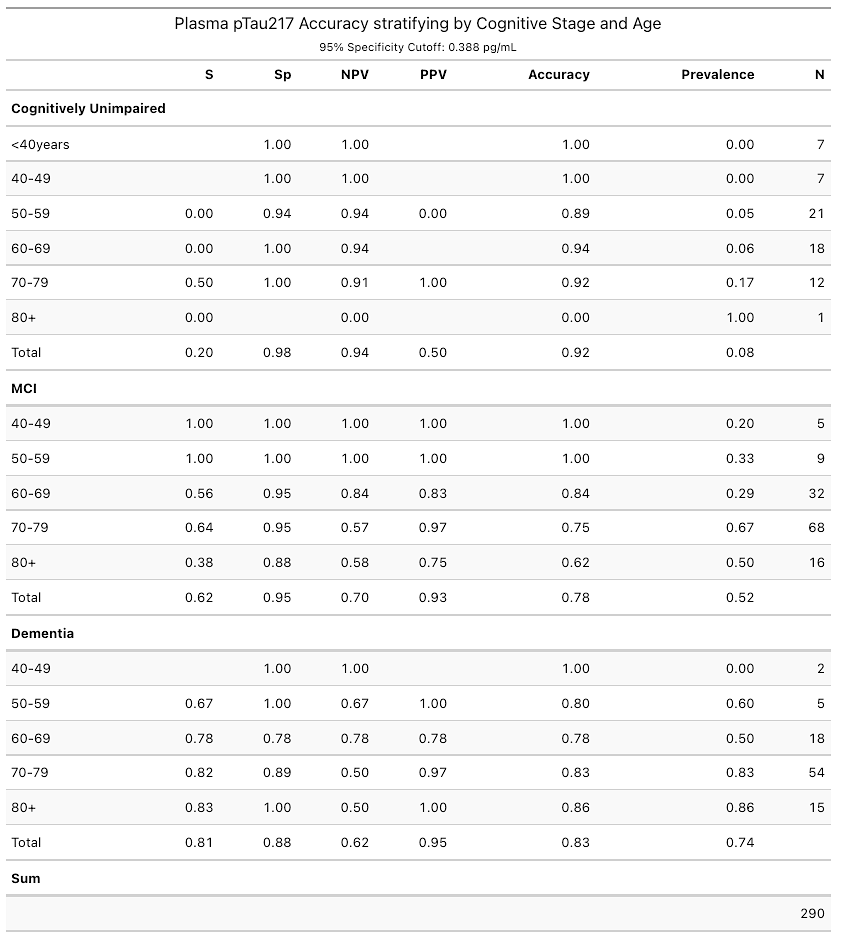


**Supplementary Table 7: Plasma pTau_217_ accuracy to detect A positivity stratifying by clinical diagnosis and age using a cutoff with a fixed sensitivity of 95%.**

CN, cognitively unimpaired; MCI, mild cognitive impairment; AD, Alzheimer disease; OtherNotDeg, other not neurodegenerative; OtherDem, other dementias. S, sensitivity; Sp, specificity; NPV, negative predictive value; PPV, positive predictive value. pTau_217_, phosphorylated tau 217.

The small number of participants with age under 60 and above 80 might alter accuracy performance for these age ranges due to potential overfitting of cutoffs in these subgroups.


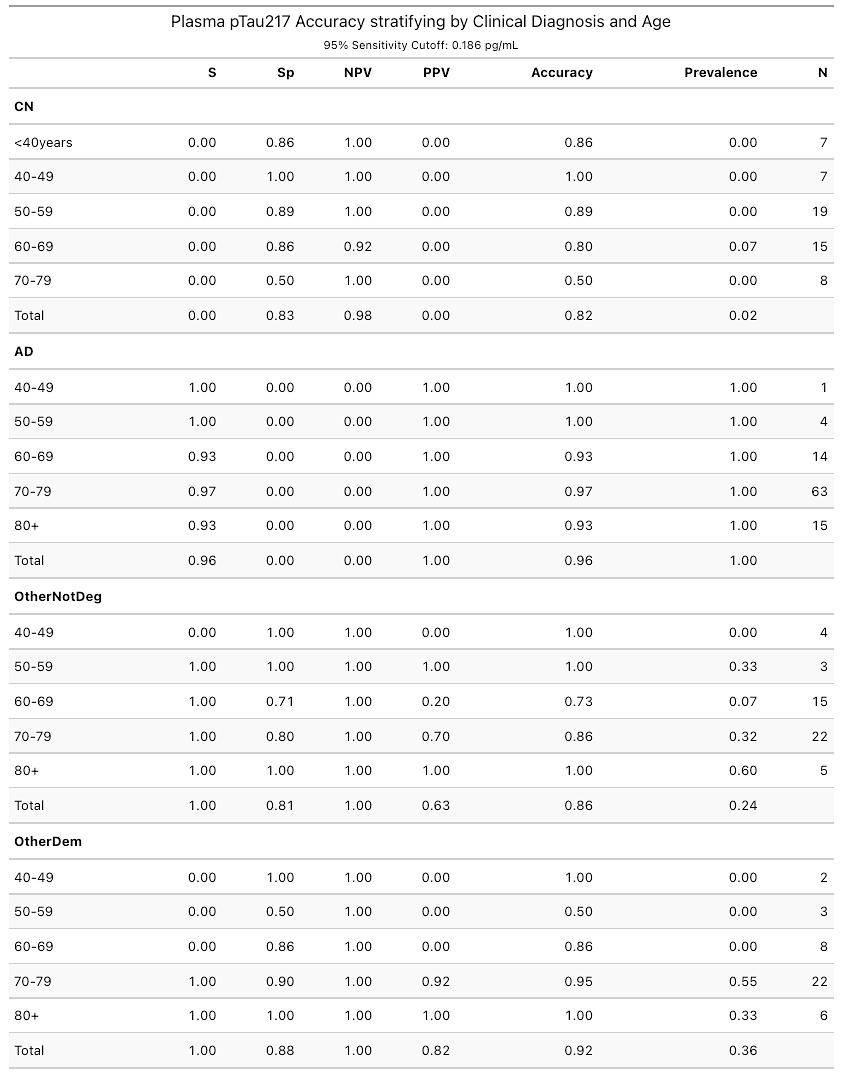

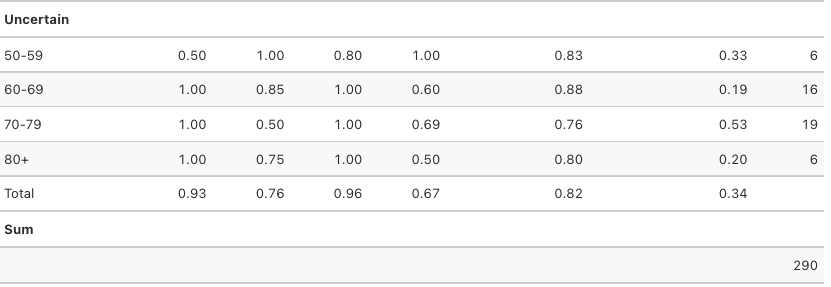


**Supplementary Table 8: Plasma pTau217 accuracy to detect A positivity stratifying by clinical diagnosis and age using a cutoff with a fixed specificity of 95%**

CN, cognitively unimpaired; MCI, mild cognitive impairment; AD, Alzheimer disease; OtherNotDeg, other not neurodegenerative; OtherDem, other dementias. S, sensitivity; Sp, specificity; NPV, negative predictive value; PPV, positive predictive value. pTau_217_, phosphorylated tau 217.

The small number of participants with age under 60 and above 80 might alter accuracy performance for these age ranges due to potential overfitting of cutoffs in these subgroups


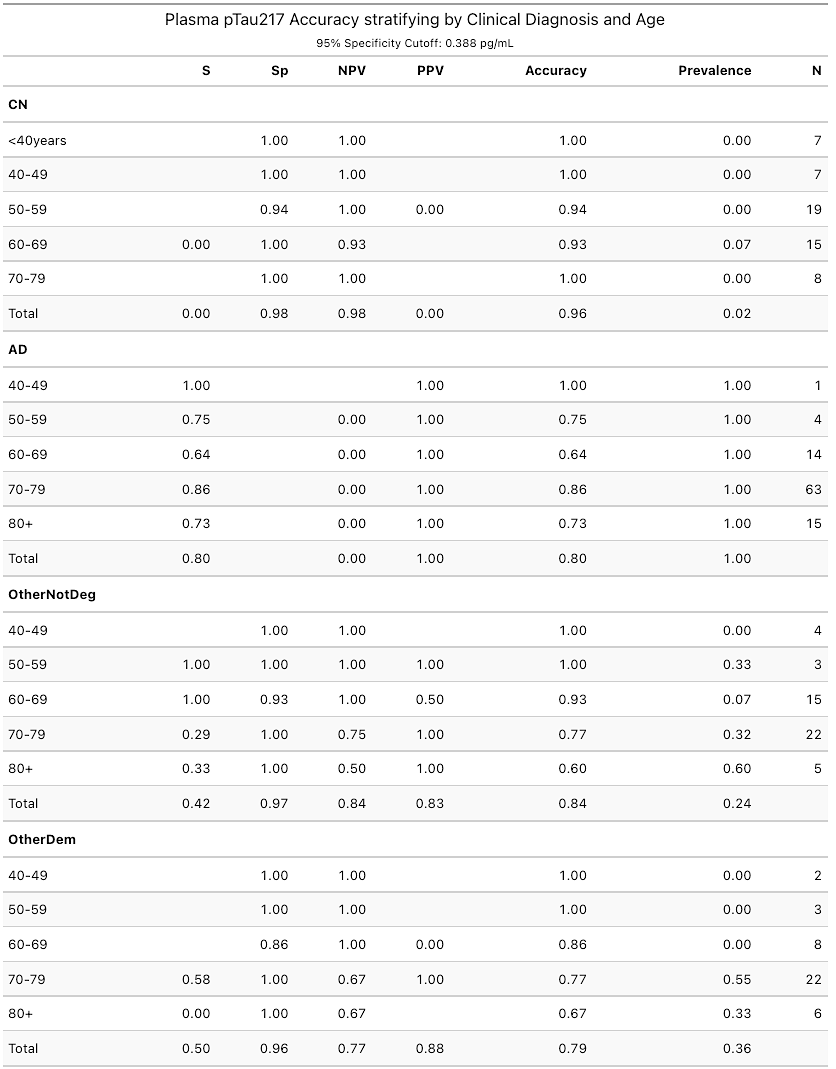

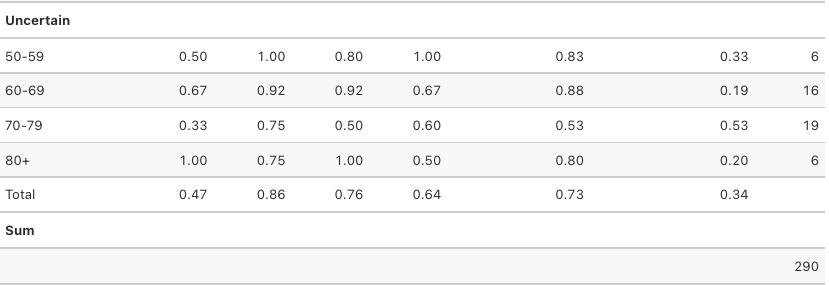


**Supplementary Figure 1: Flowchart of study participants inclusion and exclusion criteria and sensitivity analysis**

**LP, lumbar puncture; AD, Alzheimer disease; GDS, global deterioration scale; CSF, cerebrospinal fluid; CU, cognitively unimpaired; MCI, mild cognitive impairment; MMSE, minimental state examination.**

**Supplementary Figure 2: Sensitivity analysis. Plasma Biomarker concentrations’ according the CSF amyloid status.**

pTau_217_, phosphorylated tau 217, pTau_181_, phosphorylated tau 181. Aβ_1–42_, Amyloid β_1–42_. Aβ_1–40_, Amyloid β_1–40_. CU, cognitively unimpaired. MCI, mild cognitive impairment.

Plasma biomarkers Cohen’s d and Fold Change calculated in log-transformed data.

**Supplementary Figure 3: Sensitivity analysis excluding participants close to the CSF Aβ_1–42_/Aβ_1–40_ cutoff. Plasma Biomarkers Accuracy, ROC curves.**

**In this sensitivity analysis, participants with a** CSF Aβ_1–42_/Aβ_1–40_ value between 0.056 and 0.068 (0.062+/- 10%) were excluded.

pTau_217_, phosphorylated tau 217, pTau_181_, phosphorylated tau 181. Aβ_1–42_, Amyloid β_1–42_. Aβ_1–40_, Amyloid β_1–40_. CU, cognitively unimpaired. MCI, mild cognitive impairment.

**
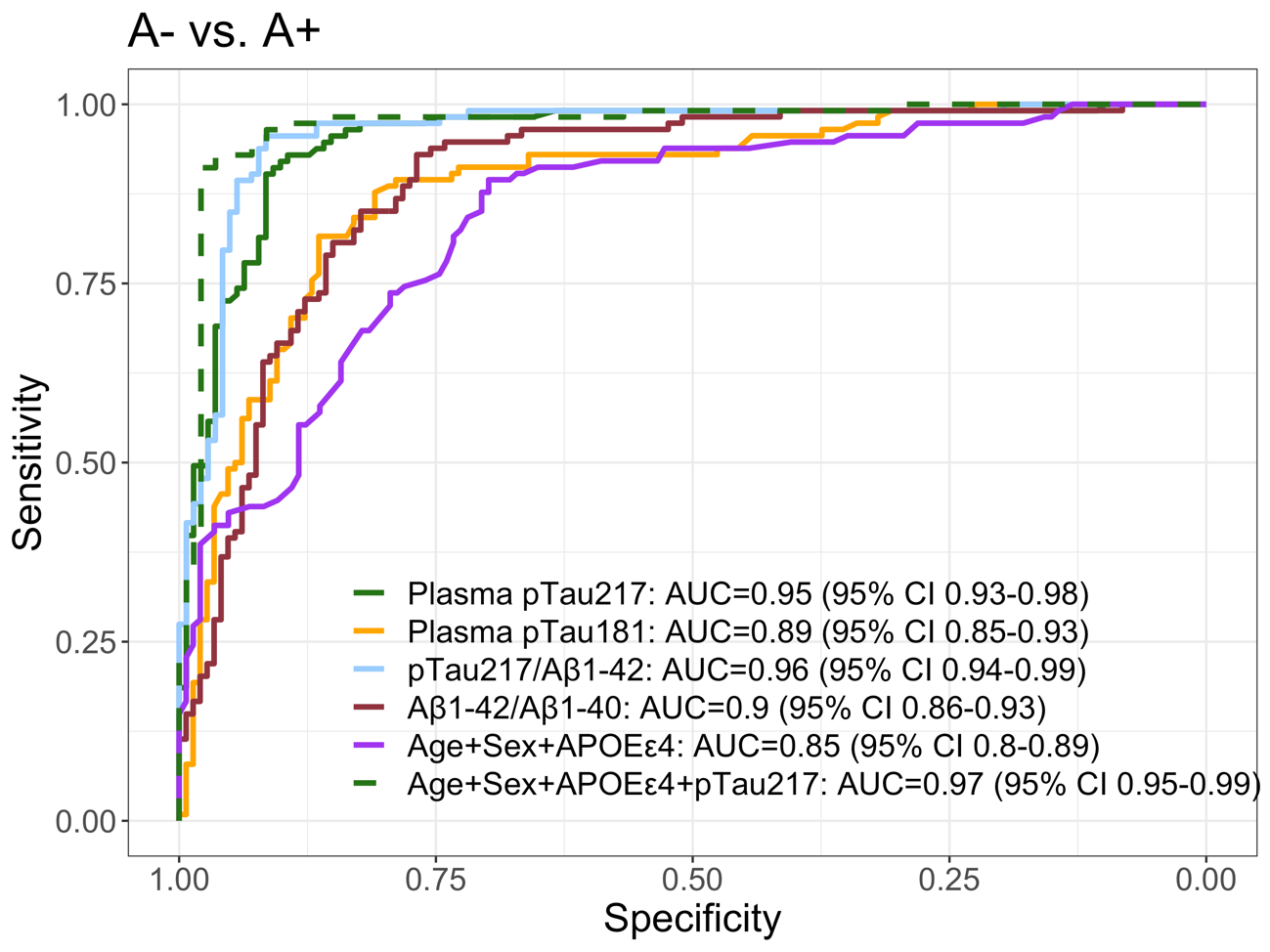
**

**Supplementary Figure 4: Correlation maps by cognitive status**

pTau_217_, phosphorylated tau 217, pTau_181_, phosphorylated tau 181. Aβ_1–42_, Amyloid β_1–42_. Aβ_1–40_, Amyloid β_1–40_. CU, cognitively unimpaired. MCI, mild cognitive impairment. Significance of the correlations was adjusted by multiple correlations (p-value adjusted by length of comparisons).


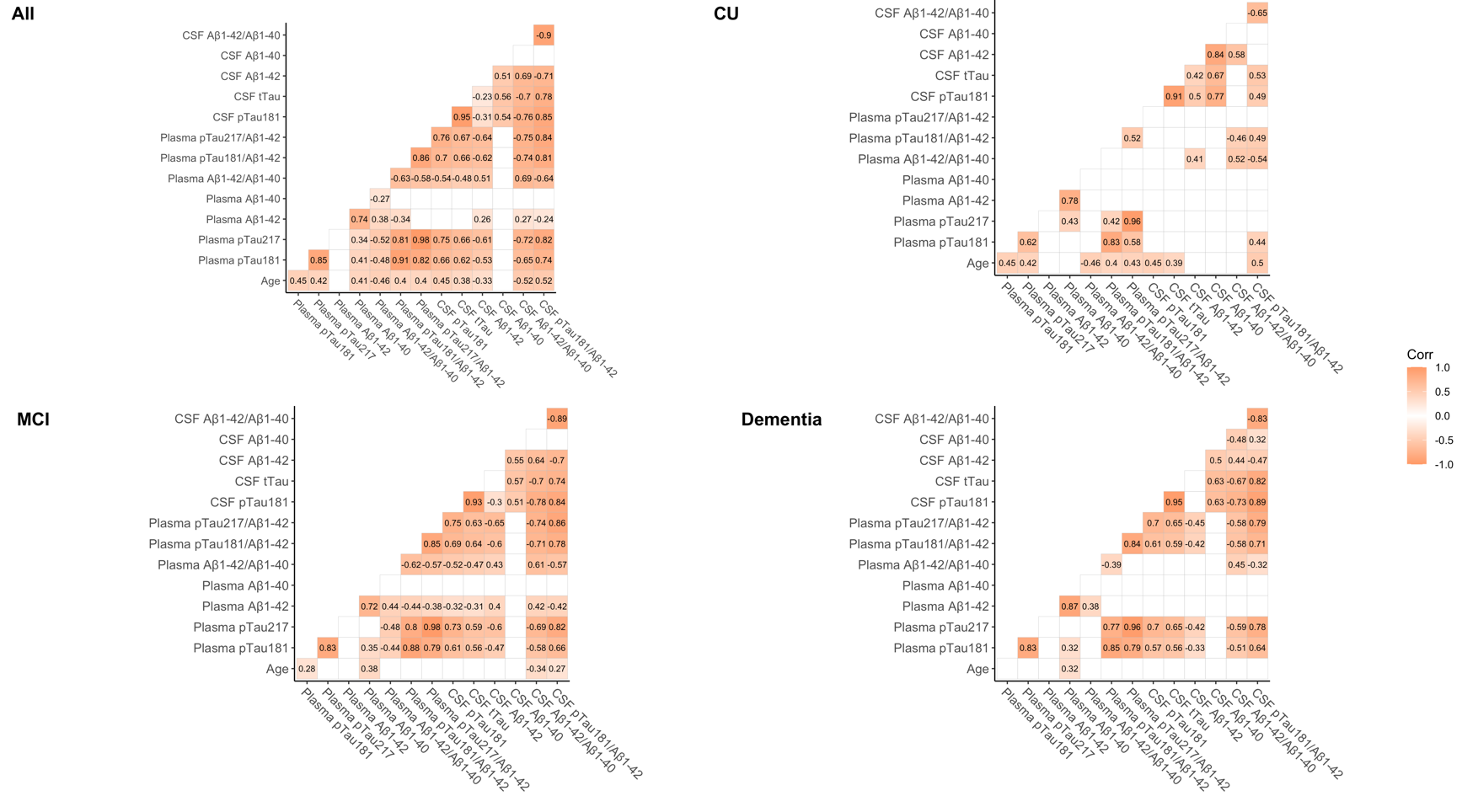


**Supplementary Figure 5: Global and partial correlations of plasma biomarkers with CSF biomarkers**

**
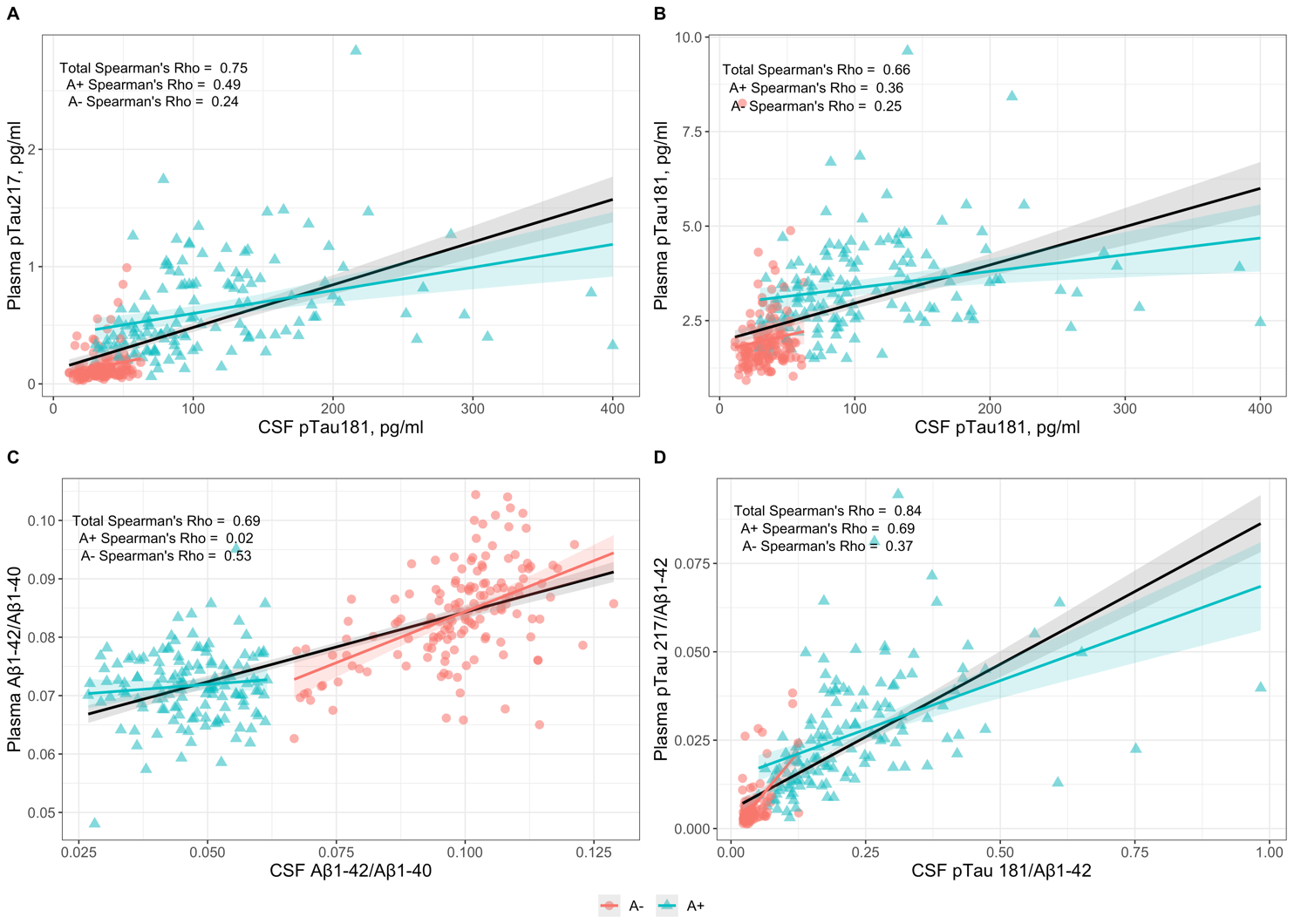
**pTau_217_, phosphorylated tau 217, pTau_181_, phosphorylated tau 181. Aβ_1–42_, Amyloid β_1–42_. Aβ_1–40_, Amyloid β_1–40_.

**Supplementary Figure 6: Complete Forest Plot of the influence of different variables in plasma biomarker concentrations.**

Dots and bars represent the standardized beta coefficients of each variable in a multivariate regression model. Lines represent the 95% confidence interval for each standardized beta coefficient. Red vertical dashed lines indicate a null effect. We can see the effect size of A positivity adjusted by other variables.

pTau_217_: phosphorylated tau 217. pTau_181_: phosphorylated tau 181. Aβ_1–42_: Amyloid β_1–42_. Aβ_1–40_: Amyloid β_1–40_. eGFR: estimated glomerular filtration rate. VRF: vascular risk factors. HBP: high blood pressure. DLP: Dyslipidemia. DM, Diabetes mellitus. OSA, obstructive sleep apnea. MCI: mild cognitive impairment.

*
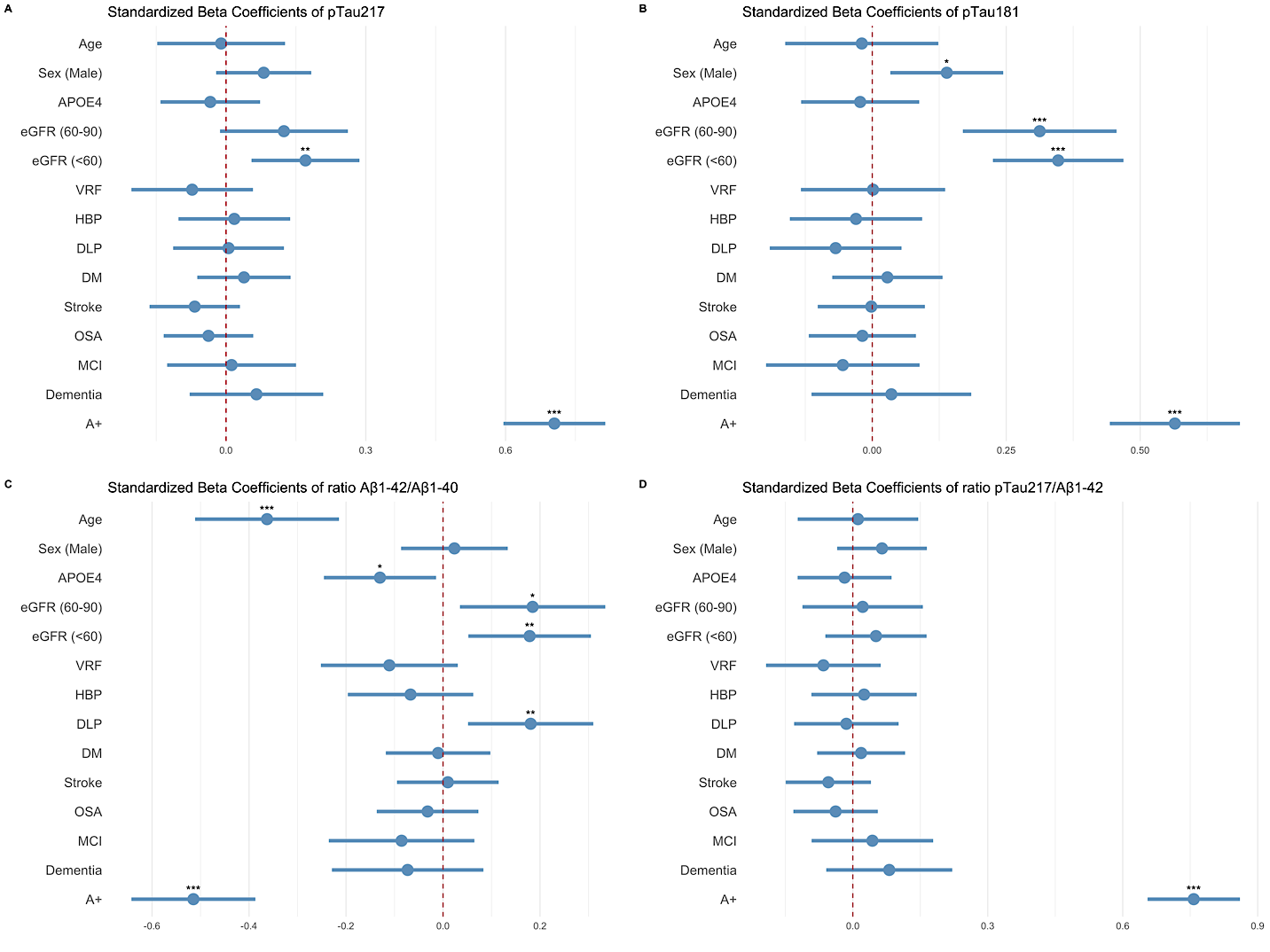
*

**Supplementary Figure 7: Diagnostic accuracy of plasma markers to detect amyloid positivity in CSF**


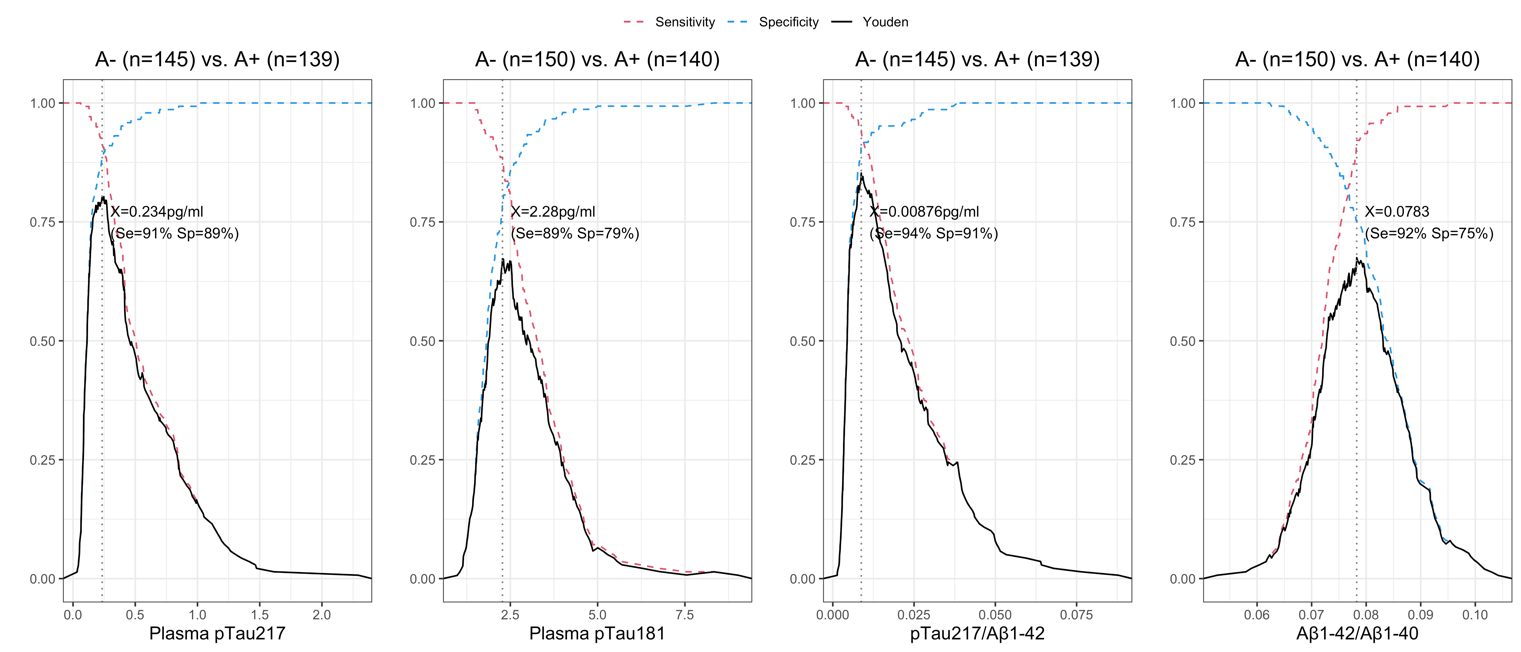
 pTau_217_, phosphorylated tau 217, pTau_181_, phosphorylated tau 181. Aβ_1–42_, Amyloid β_1–42_. Aβ_1–40_, Amyloid β_1–40._

**Supplementary Figure 8: Areas Under the ROC curve of plasma markers to detect A positivity in distinct clinical subgroups**


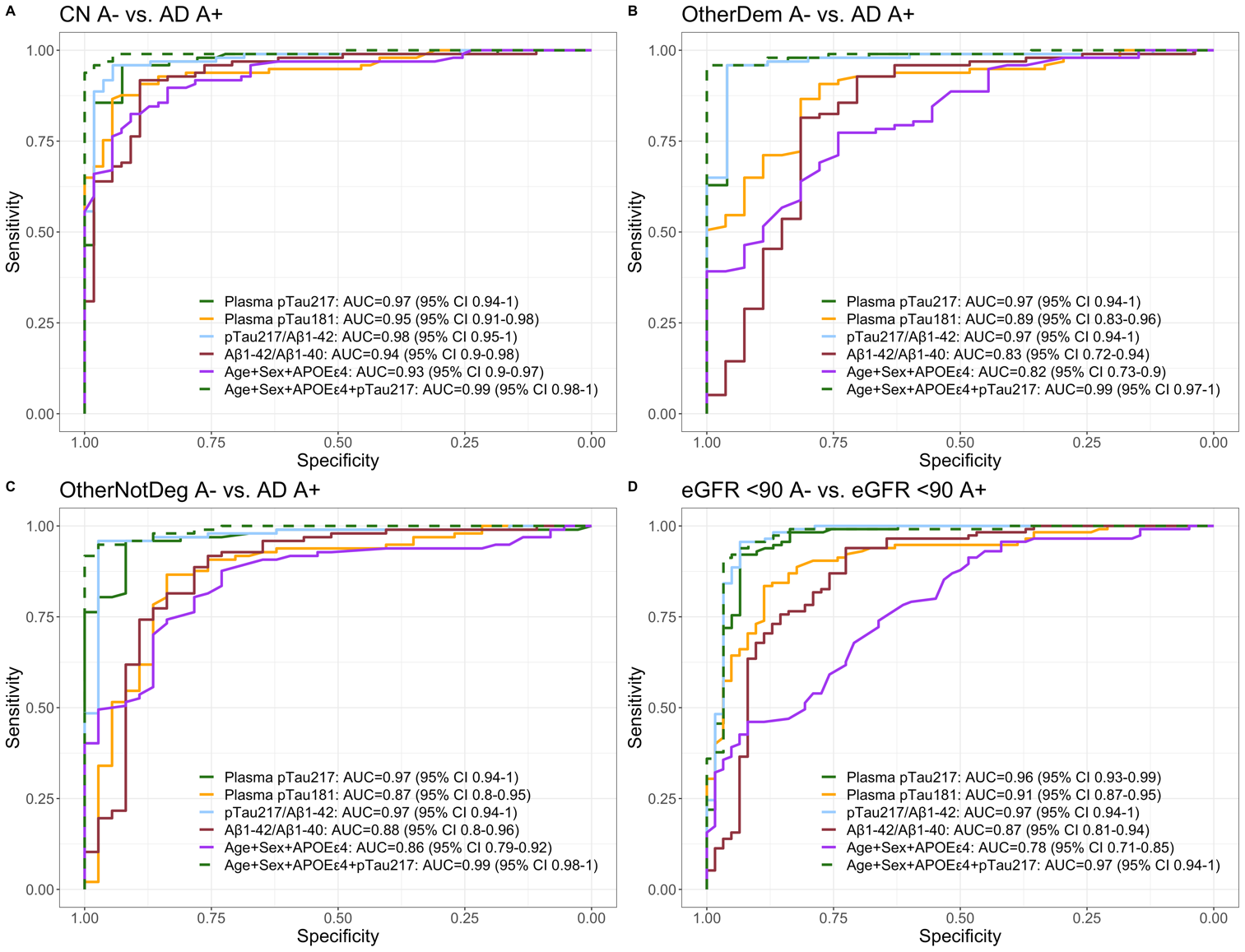
CU, cognitively unimpaired; AD, Alzheimer Disease; OtherDem, Other dementias; OtherNotDeg, Other not neurodegenerative. pTau_217_, phosphorylated tau 217. pTau_181_, phosphorylated tau 181. Aβ_1–42_, Amyloid β_1–42_. Aβ_1–40_, Amyloid β_1–40_.

**Supplementary Figure 9: Areas Under the ROC curve of plasma markers to detect the CSF A+T+ profile**

**
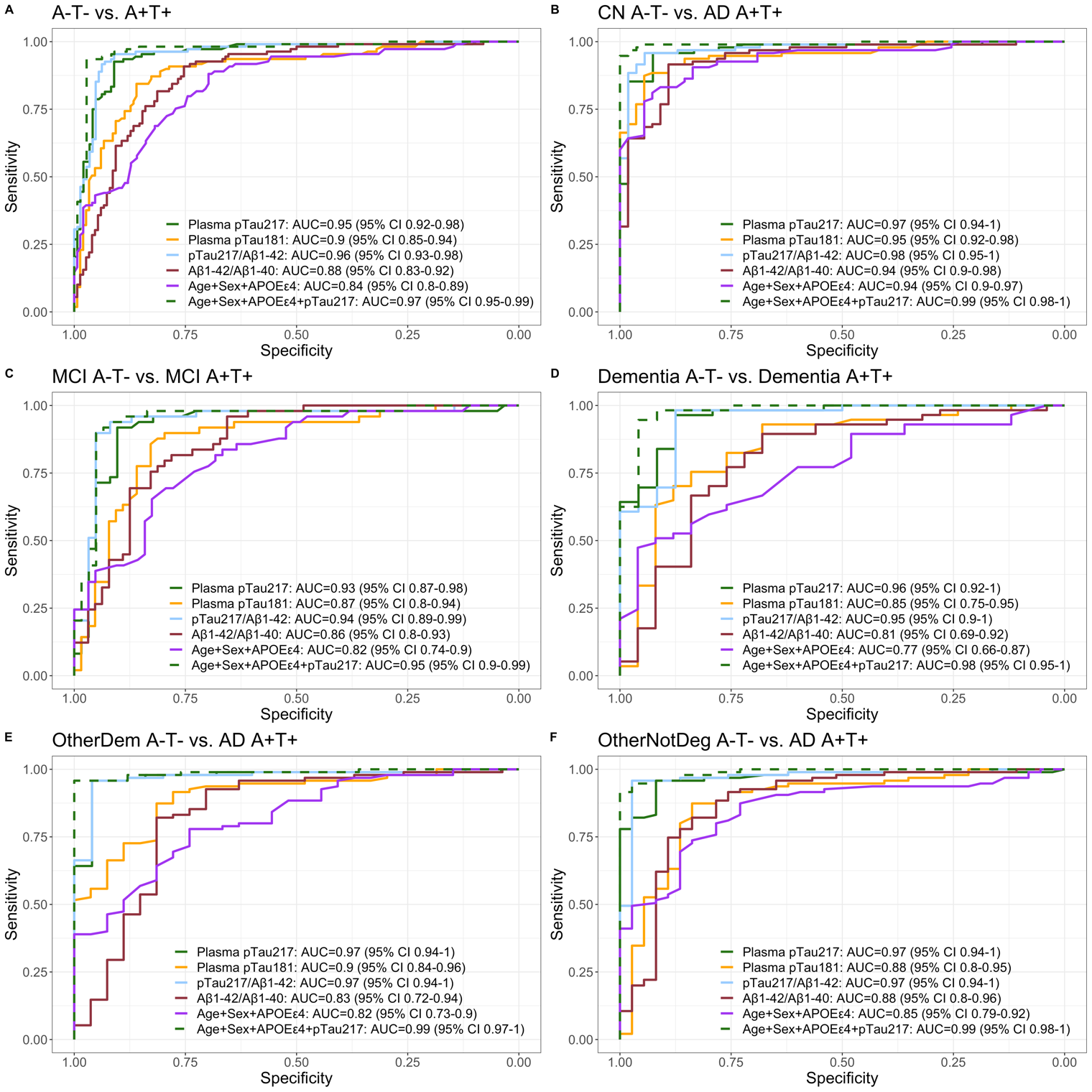
**CU, cognitively unimpaired; AD, Alzheimer Disease; OtherDem, Other dementias; OtherNotDeg, Other not neurodegenerative. pTau_217_, phosphorylated tau 217. pTau_181_, phosphorylated tau 181. Aβ_1–42_, Amyloid β_1–42_. Aβ_1–40_, Amyloid β_1–40_.

**Supplementary Figure 10: Data-driven supervised decision tree of plasma markers and demographic factors to detect amyloid positivity**

We performed a decision tree analysis, a supervised, data-driven predictive model that identified the most informative variables for predicting CSF amyloidosis. We included pTau_217_, pTau_181_, ratio Aβ_1–42_/ Aβ_1–40_, Age, Sex and *APOE ε*4. The decision tree starts with a complexity parameter (CP) of 0.80, indicating it will not make additional splits unless they can significantly reduce the relative error rate, which is a good indication of controlling overfitting. Initially, the tree splits into two child nodes, where pTau_217_ emerges as the most important variable for the first division (highest Youden cutoff 0.23 pg/mL). Subsequently, a second division is made in the right node with a CP of 0.05, using Aβ_1–42/1-40_ as the main variable for the partition (highest sensitivity cutoff 0.086). The variables pTau_217_, pTau_181_, and Aβ_1–42/1-40_ stand out as the most determinative in the model. In terms of error, the initial cross-validation error (xerror) after the first division is 0.27, compared to a relative error of 0.20, reflecting a good fit of the model to the initial data. However, the increase in cross-validation error after the first division indicates a potential onset of overfitting in subsequent divisions. The standard deviation of the cross-validation error (xstd) initially is 0.04, providing confidence in the stability of these error estimates. The misclassification rate for this algorithm was 7.4% (Sensitivity = 91%, Specificity = 94%, Accuracy = 93%, PPV = 94%, and NPV = 91%, with FNR = 9.4% and FPR=5.6%). Created with BioRender® (BioRender.com).

pTau_217p_, phosphorylated tau 217. pTau_181_, phosphorylated tau 181. Aβ_1–42/1-40_, Amyloid β_1–42_/Aβ_1–40_ ratio


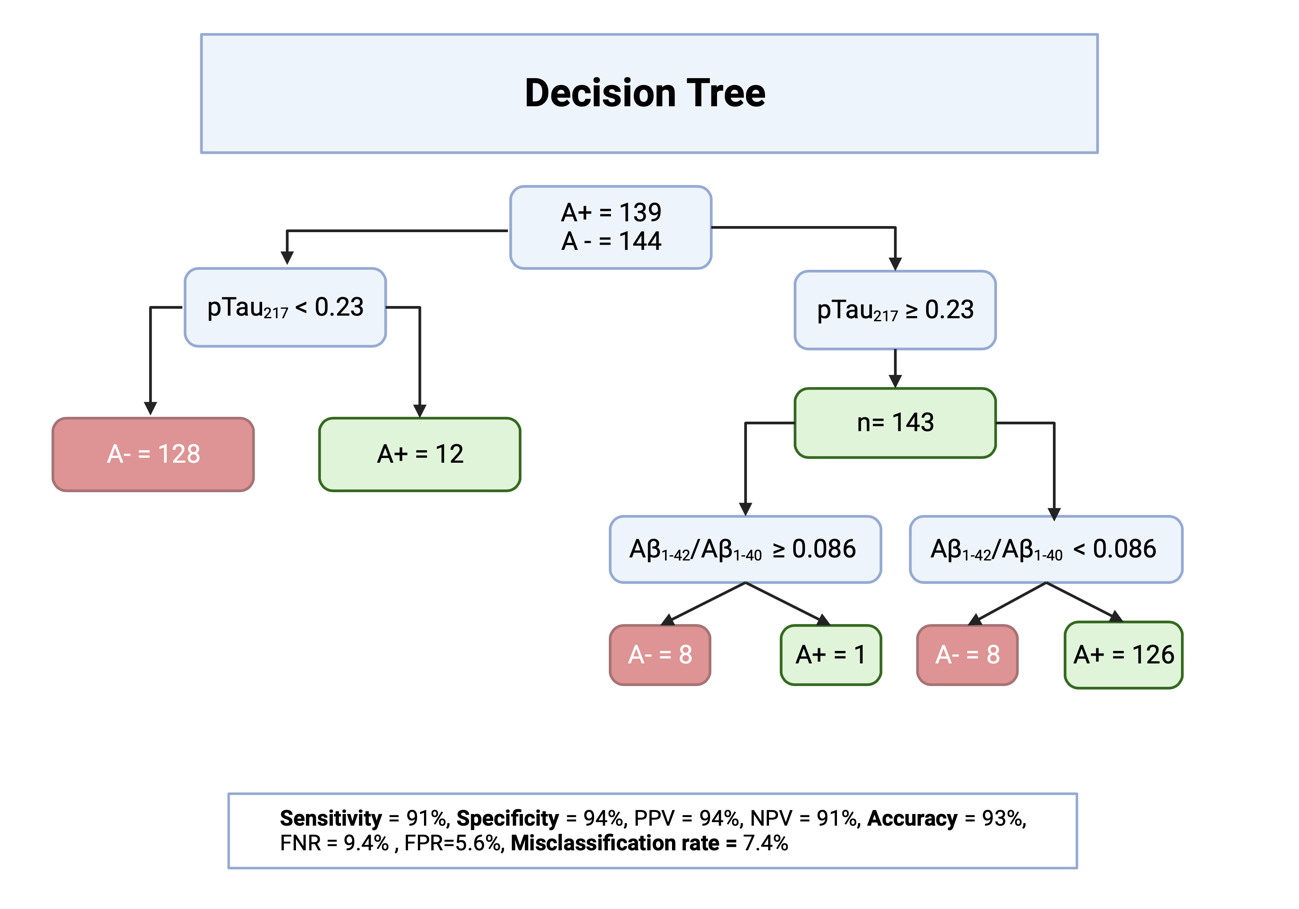

Supplement: Supplementary file 1 — Supplementary Material 1. [file 13195_2024_1513_MOESM1_ESM.docx]
